# Patient‐Derived Upper Tract Urothelial Carcinoma Organoids as a Platform for Drug Screening

**DOI:** 10.1002/advs.202103999

**Published:** 2021-12-16

**Authors:** Zhichao Li, Haibo Xu, Yanqing Gong, Wei Chen, Yonghao Zhan, Lei Yu, Yangyang Sun, Aolin Li, Shiming He, Bao Guan, Yucai Wu, Gengyan Xiong, Dong Fang, Yuhui He, Qi Tang, Lin Yao, Zheng Hu, Hongbing Mei, Zhisong He, Zhiming Cai, Yinglu Guo, Xuesong Li, Liqun Zhou, Weiren Huang

**Affiliations:** ^1^ Department of Urology Peking University First Hospital National Urological Cancer Center Beijing 100034 China; ^2^ Department of Urology Shenzhen Institute of Translational Medicine Shenzhen Second People's Hospital The First Affiliated Hospital of Shenzhen University International Cancer Center of Shenzhen University Shenzhen 518039 China; ^3^ Guangdong Key Laboratory of Systems Biology and Synthetic Biology for Urogenital Tumors Shenzhen 518035 China; ^4^ Shenzhen Institute of Synthetic Biology Shenzhen Institutes of Advanced Technology Chinese Academy of Sciences Shenzhen 518055 China

**Keywords:** drug screening, organoid, tumor heterogeneity, upper tract urothelial carcinoma

## Abstract

Upper tract urothelial carcinomas (UTUCs) are rare entities that are usually diagnosed at advanced stages. Research on UTUC pathobiology and clinical management has been hampered by the lack of models accurately reflecting disease nature and diversity. In this study, a modified organoid culture system is used to generate a library of 25 patient‐derived UTUC organoid lines retaining the histological architectures, marker gene expressions, genomic landscapes, and gene expression profiles of their parental tumors. The study demonstrates that the responses of UTUC organoids to anticancer drugs can be identified and the model supports the exploration of novel treatment strategies. This work proposes a modified protocol for generating patient‐derived UTUC organoid lines that may help elucidate UTUC pathophysiology and assess the responses of these diseases to various drug therapies in personalized medicine.

## Graphical abstract

The lack of models accurately reflecting disease nature and diversity of upper tract urothelial carcinoma (UTUC) impedes the research on its pathobiology and clinical management. This study demonstrates an organoid culture system to generate UTUC organoids from clinical samples. These organoid models retain the characteristics of their parental tumors and support the exploration of novel treatment strategies.

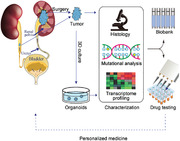

## 1 Introduction

Upper tract urothelial carcinoma (UTUC) is a rare genitourinary entity of urothelial carcinomas arising from the urothelial lining of the upper urinary tract, namely, the renal pelvis and the ureter. UTUC accounts for 5–10% of all urothelial carcinomas and 10% of all renal cancers. Its estimated annual incidence in Western countries is 2/100 000.^[^ ^1^ ^]^ Despite its low incidence, UTUC is characterized by high mortality and recurrence rates. Sixty percent and only 15–25% of all UTUC and bladder cancer cases, respectively, are invasive at diagnosis.^[^ ^1^, ^2^ ^]^ The five‐year survival rate for UTUCs invading the muscle wall is <50% at the pT2/pT3 stages and <10% at the pT4 stage.^[^ ^1^, ^3^ ^]^ Clinical UTUC management is guided by stage, grade, and location. Radical nephroureterectomy with bladder cuff excision is the standard UTUC treatment. Systemic chemotherapy is administrated for advanced UTUC.^[^ ^1b^ ^,^ ^4^ ^]^ However, adjuvant and neoadjuvant chemotherapy do not always provide survival benefit because of intra‐ and intertumor heterogeneity.^[^ ^5^ ^]^

Current UTUC neoadjuvant/adjuvant chemotherapy strategies are extrapolated from bladder cancer treatment experiences. Though UTUCs are types of urothelial tumors, their typical genomic alterations and related gene expression profiles differ from those of bladder cancers.^[^ ^6^ ^]^ Gene methylation and microsatellite instability occur more frequently in UTUCs than bladder carcinomas.^[^ ^7^ ^]^ The incidence of UTUC is increasing. Nevertheless, UTUCs remain rare malignancies and relevant human models are unavailable for them. Hence, effective in vitro models must be developed to study UTUC pathophysiology and devise effective treatment strategies for UTUCs.

Cancer cell lines and patient‐derived xenografts (PDX) are commonly used as models to clarify tumor pathophysiology mechanism and validate the efficacy of candidate therapeutic regimens. Few UTUC cell lines have been successfully established to date, however, one study reported the development of UTUC PDXs.^[^ ^8^ ^]^ Creation of these models is inefficient, labor‐intensive, and time‐consuming; hence, they are impractical for guiding personalized treatment. Moreover, the extensive, persistent presence of murine viruses in PDX models alters gene expression profiles and tumor sensitivity to treatment regimens.^[^ ^9^ ^]^ Therefore, an effective preclinical UTUC model must faithfully represent clinical manifestations and support large‐scale drug screening.

Cancer organoid models have been developed from prostate,^[^ ^10^ ^]^ colorectal,^[^ ^11^ ^]^ pancreatic,^[^ ^12^ ^]^ liver,^[^ ^13^ ^]^ breast,^[^ ^14^ ^]^ bladder,^[^ ^15^ ^]^ gastric,^[^ ^16^ ^]^ ovarian,^[^ ^17^ ^]^ and endometrial^[^ ^18^ ^]^ cancers. Two prior studies described tumor organoid generation from urothelial carcinoma.^[^ ^15^, ^19^ ^]^ Nevertheless, the studies focused mainly on culturing organoids derived from bladder cancer tissues. No optimization of organoid cultures from UTUC has been reported. Furthermore, the substantial differences in the pathophysiology of UTUCs and bladder cancers could influence the niche factor dependencies of each tumor organoid type.^[^ ^20^ ^]^ Thus, we established a culture system to derive organoids from clinical UTUC samples. We successfully generated 25 UTUC and 6 normal organoid lines. In addition, we presented extensive phenotypic and molecular characterizations of the UTUC organoid lines including their histological architecture, clinical marker expression, genomic landscape, and gene expression profiles. We also empirically demonstrated the utility of the UTUC organoid lines as drug screening models for the identification of novel therapeutic targets and the advancement of personalized medicine.

## 2 Results

### 2.1 Establishment of a Living Biobank of Patient‐Derived UTUC Organoids

Resected UTUC tissues, their corresponding normal tissues, and blood were obtained from 32 consenting patients who underwent radical nephroureterectomy or organ‐sparing surgery in the Shenzhen Second People's Hospital (Table S1, Supporting information). The male/female ratio of 2:1 is consistent with previous reports for UTUC.^[^ ^21^ ^]^ Among these patients, 22 had high‐grade tumors (pT2+), 4 had a history of bladder cancer.

**Figure 1:**
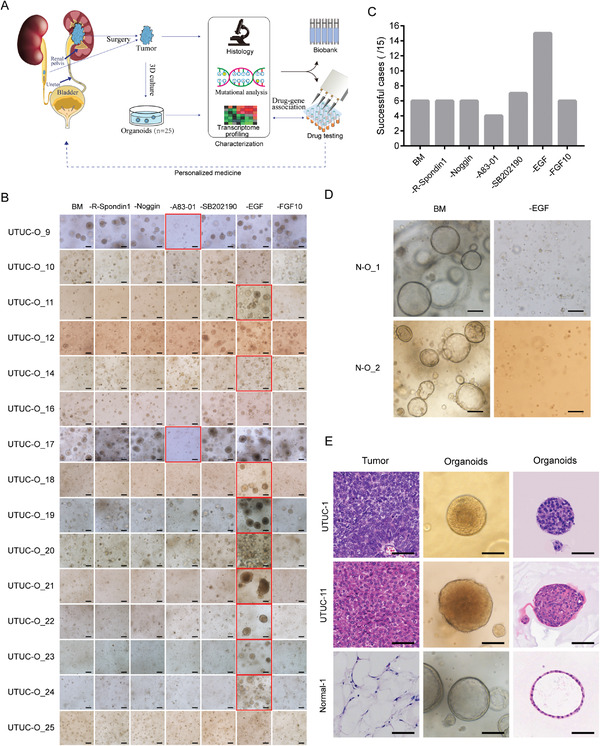
Establishment of patient‐derived UTUC organoids. A) Overview of experimental design. B) UTUC organoid formation in BM and modified medium (with each component individually omitted from the BM), shown are bright‐field images of UTUC organoids formed after 2 weeks of culture in indicated medium. Scale bar, 100 µm. C) A summary of UTUC organoid lines established in BM and modified media. D) Bright‐field images of normal tissue‐derived organoids formed after 2 weeks of culture in BM and modified medium (with EGF omitted from the BM). Scale bar, 100 µm. E) Representative H&E staining images and bright‐field images of organoids and corresponding parental tissues. Scale bar, 100 µm.

Each tissue was sectioned for organoid derivation, histological analysis, DNA extraction, and RNA isolation (**Figure** **1**A). Minced tissue samples were dissociated by a combination of mechanical dissociation and enzymatic digestion. Isolated cells were then embedded in basement membrane extract (Matrigel) and supplemented with organoid culture medium.

Based on other cancer organoid cultures, the basal medium (BM) used to culture UTUC organoids contained the Wnt agonist R‐spondin1, the transforming growth factor beta (TGF‐*β*) inhibitor Noggin, the reactive oxygen species (ROS) scavenger *N*‐acetyl‐L‐cysteine, nicotinamide, the activin receptor‐like kinase (ALK) inhibitor A83‐01, the p38 mitogen‐activated protein (MAP) kinase inhibitor SB202190, B‐27 supplement, fibroblast growth factor (FGF) 10, epidermal growth factor (EGF), and the Rho‐associated, coiled‐coil‐containing protein factor (ROCK) inhibitor Y‐27632.

UTUC organoids were not successfully generated from this culture protocol. Cell spheres appeared within the first few days but they stopped growing in some cases while others did not form at all. To test whether any culture medium components impeded UTUC organoid formation and growth, we individually omitted the foregoing components and evaluated UTUC organoid formation. The UTUC organoid lines had distinct medium component requirements. 1) EGF inhibited the formation of most UTUC organoid lines. 2) A83‐01 was essential for several UTUC organoid lines. 3) R‐spondin1, Noggin, FGF10, and SB202190 did not improve UTUC organoid formation and were excluded from the medium (Figure 1B,C). EGF was essential for the growth of organoids derived from adjacent normal tissues (Figure 1D). Under a light microscope, normal organoids appeared as hollow, cystic structures. By contrast, tumor organoids were compact and solid (Figure 1E and Figure S1, Supporting information). The composition of the media used to culture UTUC and normal organoids are listed in Table S2 (Supporting information). The first passage of UTUC organoids was conducted after 2–3 weeks of culture and the split ratio was 1:3. Organoid lines that persisted for >5 passages were considered to be successfully established.

We generated 25 UTUC organoid lines from 32 clinical UTUC samples. Moreover, six organoid lines were derived from 10 normal tissues adjacent to the tumors.

### 2.2 Histological Characterization of UTUC Organoids

To test whether UTUC organoids conserve the histological patterns of the original tumor samples, we prepared paraffin sections of them and stained them with hematoxylin‐eosin (H&E). UTUC organoids were dense and solid and had typical cancer features such as disorderly cellular arrangement (Figure 1E and Figure S1, Supporting information). By contrast, normal organoids derived from paracancerous tissues were glandular and cystic and their cells were well organized (Figure 1E). Both the UTUC organoids and their parental tumors derived from different patients had diverse histological properties (Figure 1E and Figure S1, Supporting information). The UTUC‐1_O line and its parental tumor tissue were rhabdoid and had large cells with distinct borders, prominent nucleoli, and eosinophilic cytoplasmic inclusions (Figure 1E). UTUC organoids consisted exclusively of epithelial cancer cells with no vessel elements or stromal or immune components.

**Figure 2:**
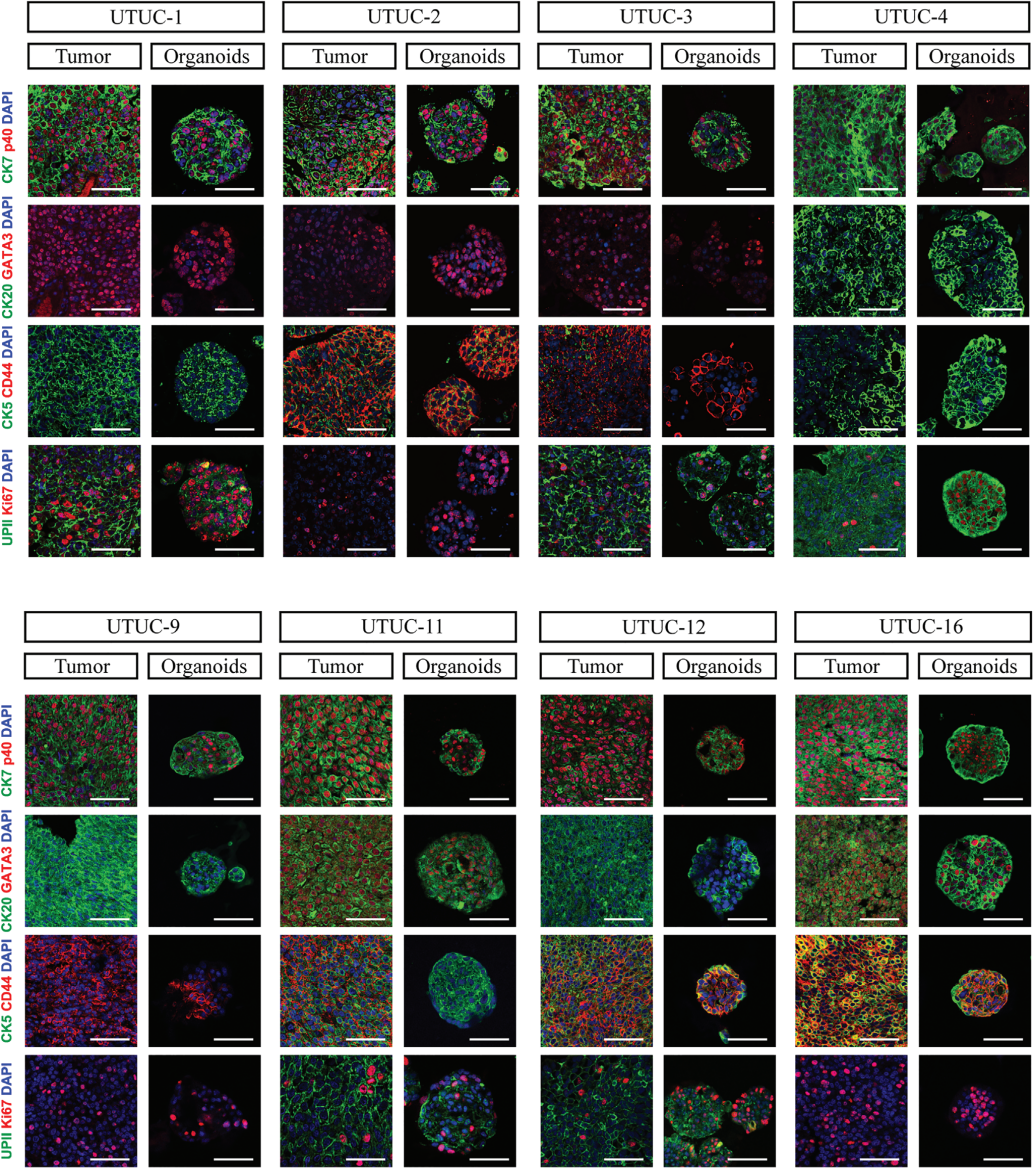
Patient‐derived UTUC organoids preserve histopathological characteristics of parental tumors. Representative immunofluorescence images of paired UTUC organoids and tumors for CK7, p40, CK20, GATA3, CK5, CD44, UP II, and Ki67. Nuclei were stained with DAPI (blue). Scale bar, 50 µm. Passage numbers of the organoid lines were: UTUC‐1_O, P6; UTUC‐2_O, P6; UTUC‐3_O, P7; UTUC‐4_O, P6; UTUC‐9_O, P8; UTUC‐11_O, P6; UTUC‐12_O, P7; UTUC‐16_O, P6.

We performed immunofluorescence analyses on eight tumor‐organoid pairs to evaluate the expression levels of their diagnostic markers. The marker expression profile of each organoid line matched that of its corresponding parental tumor (**Figure** **2**). The epithelial marker cytokeratin 7 (CK7) was highly expressed in all eight UTUC organoid lines and their source tumor tissues. All the examined samples except UTUC‐4_O and its parental tumor were positively stained for p40. This profile for UTUC‐4_O is typical in urothelial carcinoma.^[^ ^22^ ^]^ Five UTUC organoids and their corresponding tumors exhibited positive staining for the luminal marker GATA3 and the urothelial marker cytokeratin 20 (CK20) and these were conserved between the organoids and their tumor sources. UTUC‐1, UTUC‐4, UTUC‐11, UTUC‐12, and UTUC‐16 organoids and their parental tumors were positively stained for the basal epithelial marker, cytokeratin 5 (CK5). UTUC‐2 and UTUC‐3 organoids and their parental tumors were weakly stained for CK5. In UTUC‐9 organoids and tumors, however, there was no CK5 staining. The CD44 expression pattern was consistent among all organoid‐tumor pairs except UTUC‐11 whose organoids lost CD44 expression during culture. The cellular proliferation marker Ki67 was positively stained in all the samples. Hence, all organoids contained actively cycling cells. The foregoing data suggest that the differentiation hierarchies of the UTUC tissues were conserved in their corresponding organoid lines. Most (5/8) of the organoid lines and their parental tumors were positive for the newly described urothelial carcinoma marker known as uroplakin II (UP II).

### 2.3 UTUC Organoids Retain the Mutational Spectra of Their Corresponding Parental Tumors

We performed whole‐exome sequencing (WES) to determine whether UTUC organoids conserve the genetic mutations found in their parental tumors. We filtered variants and excluded polymorphisms by comparing the WES results of organoids and their parental tumors against those of matched normal blood.

**Figure 3:**
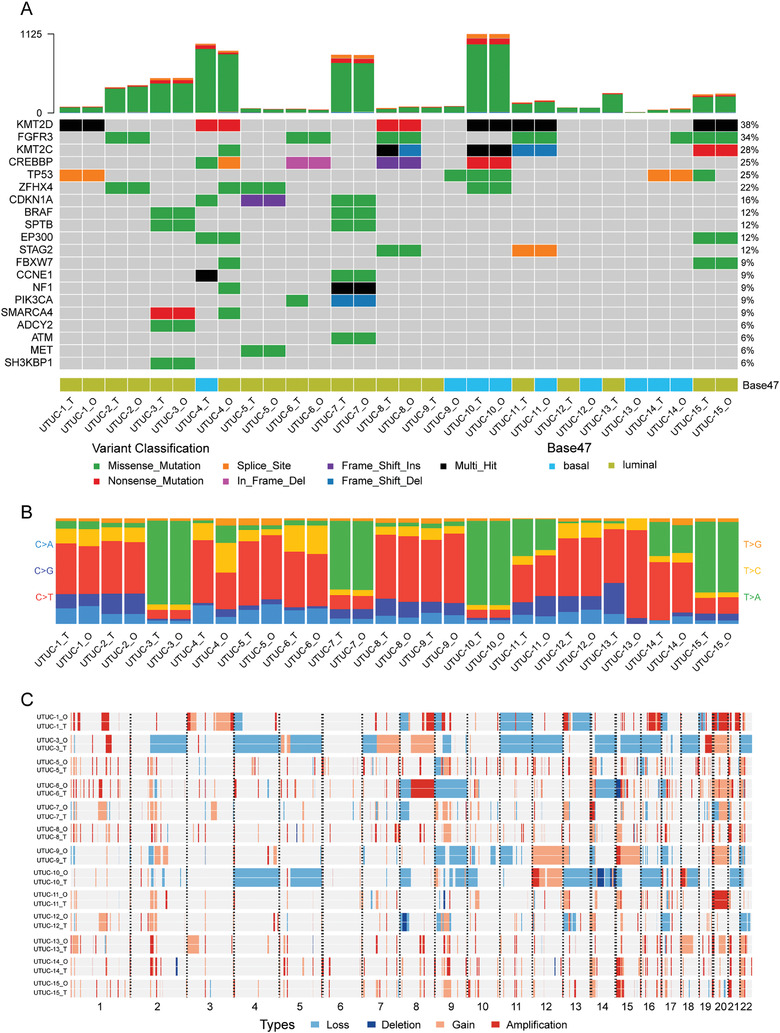
UTUC organoids recapitulate the genetic alterations in the parental tumors. A) Somatic genomic landscape of 15 UTUC organoid lines (_O) and the corresponding parental tumors (_T). The types of genetic alterations are indicated in the legend. The molecular subtypes were analyzed using the BASE47 classifier. B) Proportions of base substitutions in UTUC organoids (_O) and parental tumors (_T), the six types of base substitutions are represented. C) CNVs in UTUC organoids (_O) and tumor tissues (_T). Passage numbers of the organoid lines were: UTUC‐1_O, P5; UTUC‐2_O, P5; UTUC‐3_O, P6; UTUC‐4_O, P7; UTUC‐5_O, P6; UTUC‐6_O, P5; UTUC‐7_O, P6; UTUC‐8_O, P5; UTUC‐9_O, P7; UTUC‐10_O, P6; UTUC‐11_O, P6; UTUC‐12_O, P5; UTUC‐13_O, P6; UTUC‐14_O, P5; UTUC‐15_O, P6.

To quantify the genetic correlations between UTUC organoids and their parental tumors, we analyzed their known shared UTUC‐associated missense, splice site, and frameshift mutations, somatic base substitutions, and copy number variations (CNVs). Most somatic mutations present in the parental tumor tissues were conserved in their corresponding UTUC organoids. The most frequently occurring UTUC mutation was missense mutations in *FGFR3*.^[^ ^7^, ^23^ ^]^ It was identified in 6/15 UTUC organoid lines (**Figure** **3**A). Other UTUC‐associated somatic mutations were detected in *KMT2D*, *KMT2C*, *CREBBP*, *TP53*, *ZFHX4*, *CDKN1A*, *BRAF, SPTB, EP300*, and *STAG2*.^[^ ^7^, ^23^ ^]^ We observed co‐occurrence of *KMT2C* and *KMT2D* mutations in our cohort (Figure 3A). This discovery corroborated earlier findings on lung cancers.^[^ ^24^ ^]^

Most of these oncogenic mutations were conserved between the organoids and their parental tumors. However, some were lost or harbored in UTUC‐4, UTUC‐6, UTUC‐9, and UTUC‐14 (Figure 3A), possibly because of intratumor heterogeneity (Figures S2A–J and S3A–M, Supporting information). All mutation sites and spectra aligned between the organoids and their parental tumors. Nevertheless, the mutation sites and spectra of the UTUC‐4_O and UTUC‐13_O lines differed from those of their parental tumors (Figure 3A,B). Multiple subclones were detected in organoids and tumors (Figures S2A–J and S3A–M, Supporting information). The main tumor subclones remained unchanged during organoid processing as the variant allele frequency (VAF) and cancer cell fraction (CCF) for each subclone were similar between organoids and their parental tumors in most cases, except UTUC‐13 and UTUC‐14 (Figures S2A–J and S3A–M, Supporting information). High VAF and CCF revealed a high proportion of mutated tumor cells in both organoids and tumor tissues.

The most frequently occurring somatic base substitutions for UTUC tumor samples and organoids were T>A/A>T transversions (Tv) and C>T/G>A transitions (Ti). The least common mutation type among UTUC organoids and their parental tumors was T>G/A>C transversions (Figure S2K–L, Supporting information), These observations were consistent with the mutational spectra previously described for UTUCs.^[^ ^23b^ ^]^

The enriched pathways and biological functions of the most frequently mutated genes in UTUCs were associated mainly with genome integrity, chromatin complex, and RTK signaling (Figure S2M, Supporting information). These pathways were also enriched in bladder cancers.^[^ ^25^ ^]^ A CNV analysis disclosed similar DNA loss and gain patterns among the UTUC organoid lines and their corresponding tumors (Figure 3C).

### 2.4 Transcriptional Analysis of UTUC Organoids

**Figure 4:**
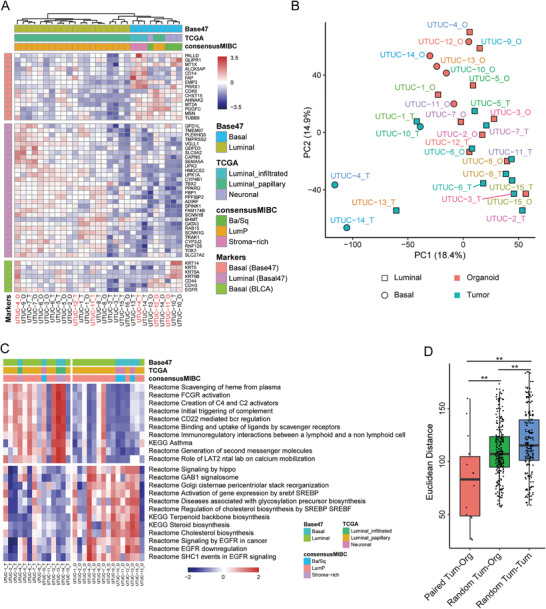
Global gene expression analysis of UTUC organoids. A) Heatmap showing molecular subtypes of UTUC organoid lines (_O) and their corresponding parental tumors (_T) based on BASE47, TCGA, and consensus MIBC classifiers. Seven genes listed on the lower panel of the heatmap were commonly used basal markers in BLCA. B) PCA plot showing UTUC organoid lines and tumor tissue distributions using top 30% variably expressed genes. C) Heatmap showing the enrichment states of the EGFR pathways and the top 10 differentially enriched pathways between tumors and organoids calculated by the GSVA algorithm with *p* < 0.01 and |GSVA_Score_Tumor_ − GSVA_Score_Organoid_| > 0.1. Pathway enrichment was calculated by GSVA algorithm. D) Euclidean distances between tumor‐organoid, random tumor‐organoid, and random tumor‐tumor pairs using the top 30% variably expressed genes. *p < 0.05, **p < 0.01. Differential analyses in C and D were performed using the Wilcoxon test. Passage numbers of the organoid lines were: UTUC‐1_O, P5; UTUC‐2_O, P5; UTUC‐3_O, P6; UTUC‐4_O, P7; UTUC‐5_O, P6; UTUC‐6_O, P5; UTUC‐7_O, P6; UTUC‐8_O, P5; UTUC‐9_O, P7; UTUC‐10_O, P6; UTUC‐11_O, P6; UTUC‐12_O, P5; UTUC‐13_O, P6; UTUC‐14_O, P5; UTUC‐15_O, P6.

We performed RNA sequencing (RNA‐seq) on 15 UTUC organoid lines and their corresponding tumor tissues. A clustering analysis using the BASE47 gene classifier grouped the samples into two molecular subtypes.^[^ ^26^ ^]^ Twenty samples displayed the luminal phenotype whereas the other nine exhibited basal phenotype (**Figure** **4**A). In most cases, the molecular subtypes were conserved between the tumor tissues and their corresponding organoids (Figure 4A). The exceptions were UTUC‐4, UTUC‐11, and UTUC‐12 (3/15). The UTUC‐11 and UTUC‐12 parental tumors expressed relatively higher luminal marker levels. By contrast, their corresponding organoids expressed mainly basal markers and had lost their luminal phenotype (Figure 4A). This discovery was in agreement with the finding for bladder cancer organoid cultures.^[^ ^15^ ^]^ The UTUC‐4 tumor tissue was clustered to the basal phenotype whereas its corresponding organoids showed a transition toward a luminal phenotype (Figure 4A). The mutational spectra also showed differences between organoids and their parental tumors (Figure 3A and Figure S2A–J, Supporting information).

Dimensionality reduction by principal component analysis (PCA) demonstrated that UTUC organoids were randomly distributed and grouped by their molecule phenotypes rather than their parental tumor tissues (Figure 4B). Most UTUC organoid lines clustered together with their parental tumors. However, the UTUC‐4, UTUC‐13 and UTUC‐14 tumor tissues were separate from their corresponding organoids and other tumor tissues (Figure 4B). Analysis of the RNA‐seq results revealed high microenvironment scores of these three tumor tissues (Figure S4A, Supporting Information), which were mainly due to the high stromal components in them (Figure S4B,C, Supporting Information). Immunohistochemistry results showed that these three tumor tissues contained large proportions of CD31‐positive endothelial cells or *α*‐SMA‐positive myofibroblasts (Figure S4D, Supporting Information).

We observed that organoids were separated not only by luminal/basal subtypes but also by organoid processing with PC1&2 components (Figure 4B). We investigated the enriched signaling pathways between UTUC organoids and their source tissues to establish whether any pathways were altered during organoid culture. In fact, several of them changed (Figure 4C). The downregulated pathways were associated mainly with immune responses such as FCGR activation and complement. The upregulated pathways were associated mainly with the Hippo signaling pathway and biosynthesis processes.

To test variance during organoid processing and variance between individual tumors, we calculated the Euclidean distances between organoids and tumors. The Euclidean distances between tumor‐organoid pairs were significantly smaller than those between random tumor‐tumor and tumor‐organoid pairs (Figure 4D). Hence, there was less organoid processing than individual variation.

### 2.5 Drug Responses of UTUC Organoids

To evaluate the utility of UTUC organoids as preclinical models assessing tumor responses to anticancer drugs, drug screenings were performed on 12 UTUC organoid lines. UTUC organoids were dissociated into small clusters and plated in low‐attachment 96‐well plates in 2% Matrigel/growth medium. The organoids were treated with drugs 1 day after plating and incubated for 6 d before the cells were enumerated with Cell Titer‐Glo 3D reagent. The sensitivity of each UTUC organoid line to 24 anticancer drugs was determined by dose titration assays with technical and biological triplicates. Standard chemotherapy drugs for urothelial carcinomas and targeted agents against signaling pathways or molecules of interest were selected for this study. Several drugs undergoing validation in clinical trials were included as well. The responses of UTUC organoid lines to the drugs were quantitated using half‐maximal inhibitory concentrations (IC_50_) and areas under the dose‐response curve (AUC).

**Figure 5:**
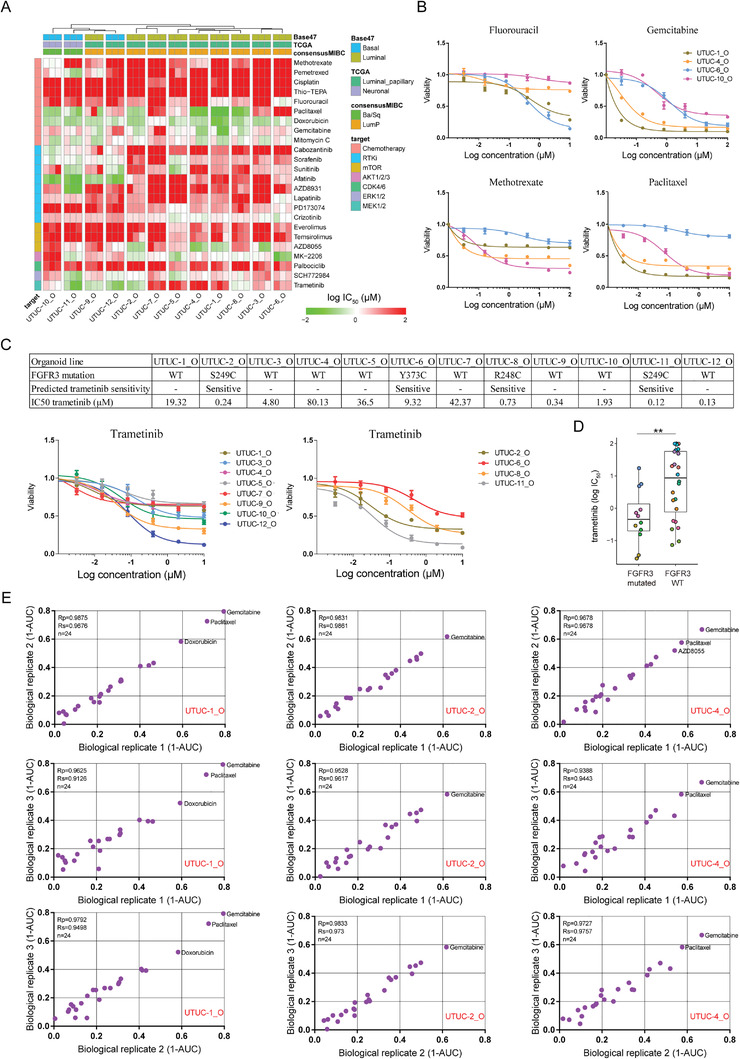
Drug screening in patient‐derived UTUC organoids. A) Heatmap of logIC_50_ values for 24 compounds versus 12 UTUC organoid lines. Columns were clustered based on the average logIC_50_ values using the ward.D2 algorithm in R. Red and green color represented the logIC_50_ value. Bright red represented logIC_50_ > 2 (100 × 10^−6^ m) and bright green represented logIC_50_ <−2 (0.01 × 10^−6^ m). B) Dose‐response curves of UTUC organoid lines treated with the indicated drugs. Each data point represents the mean of values from three biological replicates. C) Genetic alterations in *FGFR3* in UTUC organoid lines and dose‐response curves of *FGFR3* wildtype UTUC organoid lines (left) and *FGFR3* mutant organoid lines (right) treated with trametinib. D) Boxplot showing the logIC_50_ values of trametinib on *FGFR3* mutated and wildtype UTUC organoid lines using Wilcoxon test. Each dot represents one biological replicate of one organoid line. **p < 0.01. E) Representative scatterplots of 1‐AUC for two biological replicates (organoids from different passages) of the drug screening data. Drugs highlighted in red had substantial inhibitory effect on the viability of indicated organoid lines (1‐AUC > 0.5 for both biological replicates).

Most UTUC organoids were sensitive to gemcitabine. Combinations of this drug with other agents are recommended for first‐line UTUC treatment (**Figure** **5**A,B). However, UTUC‐7_O, UTUC‐10_O, and UTUC‐12_O, were resistant to gemcitabine. Hence, patient‐derived cancer organoids must be used to assess drug responses.

Drug responses markedly differed among UTUC organoid lines (Figure 5A,B and Figure S5, Supporting information). UTUC‐6_O line was sensitive to fluorouracil but resistant to methotrexate and paclitaxel whereas UTUC‐10_O line was resistant to fluorouracil but sensitive to methotrexate and paclitaxel (Figure 5A,B). UTUC‐1_O was highly sensitive to gemcitabine and paclitaxel while UTUC‐4_O was highly sensitive to gemcitabine alone (Figure 5A,B). Interestingly, luminal and basal UTUC organoids showed differential responses to some drugs such as gemcitabine and trametinib (Figure 5A and Figure S6, Supporting information).

The combination of UTUC organoid lines mutational spectra and drug responses revealed that UTUC organoids could serve as platforms for identifying efficacious targeted therapies. Several UTUC organoid lines harbored *FGFR3* mutations. These lines included UTUC‐2_O (FGFR3 S249C), UTUC‐6_O (FGFR3 Y373C), UTUC‐8_O (FGFR3 R248C), UTUC‐11_O (FGFR3 S249C), UTUC‐14_O (FGFR3 Y373C), and UTUC‐15_O (FGFR3 S249C). UTUC‐2_O, UTUC‐8_O, and UTUC‐11_O were highly sensitive to the MEK inhibitor trametinib whereas UTUC‐6_O was relatively less sensitive to it (Figure 5C). The *FGFR3* wildtype UTUC‐12_O line was highly responsive to trametinib treatment (Figure 5C). Wilcoxon test revealed that *FGFR3* mutated UTUC organoids are more sensitive to trametinib (Figure 5D). The molecular mechanism underlying the differences in trametinib response among the foregoing organoid lines remains unclear. Thus, it is useful to combine WES with in vitro drug screens on UTUC organoids.

A positive correlation was identified between biological replicate runs (the same organoid line, different passages). Therefore, the drug responses of these organoids were stable during organoid culture (Figure 5E and Figure S7, Supporting information).

### 2.6 scRNA‐seq Revealed the Cellular Heterogeneity of UTUC Organoids in Response to Gemcitabine

**Figure 6:**
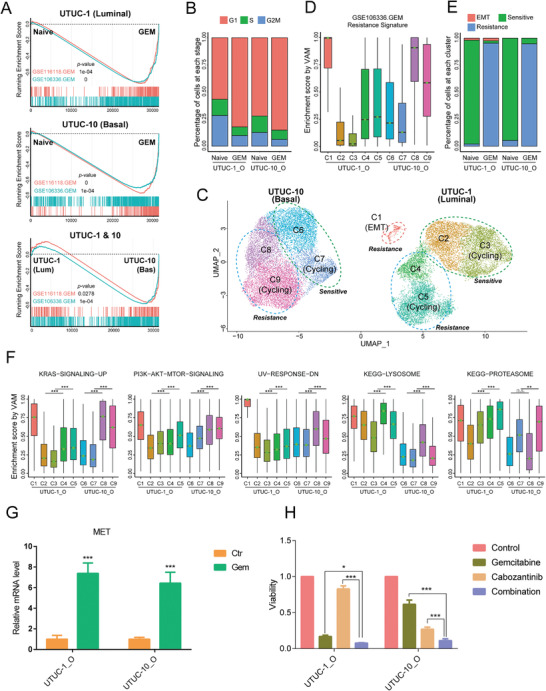
scRNA‐seq identification of cell subpopulation transition during GEM treatment. A) GSEA plot showing the enrichment state of two GEM‐resistance signatures between samples (GEM‐naive UTUC‐1_O vs GEM‐treated UTUC‐1_O; GEM‐naive UTUC‐10_O vs GEM‐treated UTUC‐10_O; and GEM‐naive UTUC‐1_O vs GEM‐naive UTUC‐10_O). B) Histogram showing the proportions of cells at each cell cycle phase (G1, S, and G2/M) and in each subcluster. C) UMAP plot showing the subclusters in UTUC‐1_O and UTUC‐10_O before and after GEM treatment. Each dot represents a single cell colored by its cluster identity. D) Boxplot showing the GSE106336 enrichment score. GEM‐resistance signature in each subcluster. E) Histogram showing the proportions of EMT, GEM‐sensitive, and GEM‐resistant cells in each subcluster. F) Boxplot showing the enrichment scores of several pathways in each subcluster. Differential enrichment analysis was performed using Wilcoxon rank sum test. G) qPCR showing the c‐MET expression levels in UTUC‐1_O and UTUC‐10_O after GEM treatment. Each data point represents three biological replicates. H) Cell viability analysis of the UTUC‐1_O and UTUC‐10_O lines after GEM (0.1 × 10^−6^ m) or cabozantinib (5 × 10^−6^ m) treatment or their combination. Each data point represents three biological replicates. The statistical differences between groups were analyzed using unpaired two‐tailed *t*‐test. Error bars represent ± SEM. *p < 0.05 and ***p < 0.001.

Gemcitabine (GEM) plus cisplatin (GC) is the first‐line chemotherapy for UTUCs. However, the resistance of tumor cells to GEM lowers treatment efficacy. To study the mechanism of GEM resistance in UTUC, we used scRNA‐seq to explore gene expression and cellular heterogeneity in the organoids. After quality control, 26 368 cells from UTUC‐1_O (luminal subtype) and UTUC‐10_O (basal subtype) were analyzed before and after GEM treatment (Figure S8A, Supporting information). Gene set enrichment analysis (GSEA) showed that the GEM‐resistance signature was significantly enriched in both organoid lines after GEM treatment (**Figure** **6**A), suggesting that GEM‐treated organoids acquired resistance to this drug. This was further proved by our observation that organoids regrown from cells survived one‐week GEM treatment demonstrated higher resistance to this drug compared with GEM‐naive organoids (Figure S9, Supporting information). Moreover, the signature genes associated with GEM‐resistance were significantly upregulated in GEM‐naive (before GEM treatment) UTUC‐10_O compared with those in GEM‐naive UTUC‐1_O. These findings were consistent with previous drug screening results demonstrating that UTUC‐10_O had a higher IC_50_ value for GEM than UTUC‐1_O (Figure 5A). The foregoing data suggest that both organoid lines became resistant to GEM after being treated with it. The basal subtype UTUC‐10_O was more resistant to GEM than UTUC‐1_O. Cell cycle analysis showed that the GEM treatment reduced the proportion of cycling cells. This result was consistent with the mechanism of GEM, namely, cell cycle arrest and inhibition of DNA synthesis (Figure 6B and Figure S8B,C, Supporting information).

Total cells were segregated into nine main clusters by dimensionality reduction and clustering analysis (Figure 6C). Subclusters C1–C5 were identified in the luminal type UTUC‐1_O line. Subclusters C6–C9 were observed in the basal type UTUC‐10_O line (Figure 6C and Figure S8D,E, Supporting information). The clusters from the luminal and basal molecular subtypes did not overlap. Thus, the UTUC subtypes had distinct gene expression profiles. The cells in C2, C3, C6, and C7 were derived mainly from GEM‐naive organoids and while those in C4, C5, C8, and C9 originated primarily from GEM‐treated organoids (Figure S8D,E, Supporting information).

The GSEA showed that the GEM‐resistance signature was more highly enriched in C1, C4, C5, C8, and C9 than it was in the parental GEM‐naive organoids (Figure 6D and Figure S8F,G, Supporting information). Hence, C2, C3, C6, and C7 were GEM‐sensitive whereas C1, C4, C5, C8, and C9 were GEM‐resistant. We found that a few cells from GEM‐naive organoids were grouped into the GEM‐resistant clusters in both organoid lines (Figure S8D,E, Supporting information). The GSEA showed that the genes in the cells of the GEM‐resistant clusters were more upregulated than they were in cells of the GEM‐naive clusters (Figure S10A, Supporting information). Therefore, a few drug‐tolerant cells were already present in the UTUC organoids before GEM treatment.

We then studied cell transition over GEM treatment using scVelo software,^[^ ^27^ ^]^ the data showed tumor cells were originated from the GEM‐resistant cluster which existed in both naive organoids or GEM‐treated organoids (Figure S11, Supporting information). There were two branches in tumor cell transition. One branch was cells from the cycling GEM‐resistant cluster transited into noncycling GEM‐resistant cluster, the other was cells from the cycling GEM‐resistant cluster transited into cycling GEM‐sensitive cluster (Figure S11A,B, Supporting information). Data from GEM‐naive organoids or GEM‐treated organoids alone showed similar results (Figure S11C–F, Supporting information). The transition originating from GEM‐tolerant cluster suggested the GEM resistance signature may not due to transcriptional reprogramming triggered by GEM, but expansion of pre‐existing drug tolerant cells.

In a special cluster, C1, the number of cells did not significantly change after GEM treatment. Moreover, C1 was highly enriched in terms of the GEM‐resistance signature (Figure S8F,G, Supporting information). Further analysis demonstrated that C1 strongly expressed epithelial‐mesenchymal transition (EMT)‐associated genes such as *VIM, ZEB2*, and *COL1A2*. It was also highly enriched with EMT transition gene sets (Figure S10B–D, Supporting information). Hence, these cells were in the EMT state and could acquire resistance to GEM as EMT progresses.

Taken together, the foregoing findings indicate that GEM‐sensitive and GEM‐resistant cells were present in both the luminal and basal UTUC subtypes and the proportion of GEM‐sensitive cells decreased after GEM treatment (Figure 6E).

We detected tumor cell heterogeneity in both GEM‐sensitive and GEM‐resistant clusters from UTUC‐1_O and UTUC‐10_O (Figure 6C). The C2 and C3 subpopulations were found in the GEM‐sensitive groups of UTUC‐1_O. We also noticed that G2M‐phase mitosis markers such as MKI67 and TOP2A were highly enriched in C3, C5, C7, and C9 both before and after GEM treatment (Figure S8B, Supporting information). These clusters were also enriched with G2M‐phase cells (Figure S8C, Supporting information). The preceding clustering results suggest the presence of actively replicating cell subpopulations before and after GEM treatment.

### 2.7 scRNA‐seq Revealed Gemcitabine Resistance Mechanism in UTUC

In both UTUC organoid lines, we measured the expression levels of candidate genes putatively responsible for GEM‐resistance (Figure S10D, Supporting information).^[^ ^28^ ^]^ Most genes were upregulated in luminal and basal GEM‐resistant clusters as well as the EMT cluster. A few were upregulated in specific clusters (Figure S10D, Supporting information). After GEM treatment, the expression levels of most genes remained unchanged except those associated with GEM metabolism, EMT, and apoptosis such as *RRM2, CDA, CDH1*, and *BID* (Figure S10D, Supporting information).

We then analyzed differentially expressed genes (DEGs) and pathways related to GEM‐resistance. The upregulated genes in the GEM‐resistant clusters of luminal or basal organoid lines were grouped into four modules (Figure S12A, Supporting information). The genes in module P1 were associated with hypoxia, TNFA signaling via NF*κ*B, EMT, p53, KRAS signaling, and mTORC1 signaling, and they were upregulated mainly in the basal type GEM‐resistant clusters. The genes in module P2 were upregulated in luminal type GEM‐resistant clusters and were associated with apoptosis, TNFA signaling via NF*κ*B, lysosomes, and so on. The genes in module P3 were upregulated in both the luminal and basal type GEM‐resistant clusters and were involved in TNFA signaling via NF*κ*B, p53, apoptosis, mTOR1, lysosomes, pyrimidine metabolism, UV response, and so on. Though modules P1–P3 had distinct genes, their enriched signaling pathways were similar. The data suggested the luminal and basal UTUC organoids acquired resistance to GEM through similar biological pathways.

Certain signaling pathways were enriched in either the luminal or basal organoids after GEM treatment. The KRAS, mTOR, and UV response pathways were upregulated in the basal type GEM‐resistant clusters (Figure 6F). The lysosome and proteasome pathways were upregulated in the luminal type GEM‐resistant clusters (Figure 6F and Figure S13A, Supporting information). GEM resistance‐associated pathways were upregulated in C1 (EMT). For this reason, the cells in C1 were GEM‐tolerant.

The basal type organoid line UTUC‐10_O was more tolerant to GEM than the luminal type organoid UTUC‐1_O (Figures 5A and 6A). Therefore, we studied the DEGs between GEM‐naive UTUC‐10_O and GEM‐naive UTUC‐1_O. The module P1 genes were constitutively upregulated in GEM‐naive UTUC‐10_O compared with GEM‐naive UTUC‐1_O. Moreover, their expression levels remained unchanged after GEM treatment. However, the genes in module P2 were upregulated after GEM treatment (Figure S12B, Supporting information). The enrichment analysis showed that GEM resistance‐associated pathways such as MYC targets v1, mTORC1, xenobiotic metabolism, and EMT, were upregulated in GEM‐naive UTUC‐10_O compared with GEM‐naive UTUC‐1_O.

Tumor cells that persist after chemotherapy treatment are reservoirs for tumor relapse and are, therefore, important barriers to good clinical outcomes. Therefore, it is vital to understand the effects of residual cancers after treatment. The scRNA‐seq data showed that several pathways and/or molecules were activated after GEM treatment (Figure S13B, Supporting information). qPCR confirmed that c‐Met mRNA was upregulated in UTUC‐1_O, UTUC‐10_O, and four other UTUC organoid lines after GEM treatment (Figure 6G and Figure S14, Supporting information). The UTUC‐1_O organoid line was resistant to the c‐Met inhibitor cabozantinib (Figure 5A and Figure S5, Supporting information). We hypothesized that a combination of cabozantinib and gemcitabine might overcome drug resistance and improve therapeutic efficacy. A cell viability assay on these two organoid lines showed that the combination of gemcitabine and cabozantinib dramatically reduced cell viability (Figure 6H). These data demonstrated that an efficacious treatment strategy can be designed for individualized therapy by combining organoid‐based drug testing with transcriptome technology.

## 3 Discussion

UTUCs are carcinomas of the upper urothelial tract including the renal pelvis and the ureter. Current treatment approaches for UTUCs were adapted mainly from bladder cancer therapies. However, there are substantial pathophysiological differences between UTUCs and bladder cancers and those may influence therapeutic efficacy.^[^ ^20^ ^]^ The overall five‐year survival rate for UTUCs is low especially at the advanced stages. Patients with UTUCs and other rare malignancies are disenfranchised as clinical trials are difficult to perform on them. Moreover, the lack of appropriate research models for this disease impedes the development of efficacious therapeutic strategies.

In this study, we established a system for the derivation and long‐term culture of UTUC organoids. We discovered that several niche factors such as R‐spondin1, Noggin, and SB202190 were not essential for UTUC organoid growth despite the fact that these substances apparently sustained organoids derived from several other cancer types. Furthermore, EGF severely hindered UTUC organoid establishment even though this compound was widely used to promote the growth of other cancer organoids. The RTK/RAS/PI(3)K pathway was highly activated in urothelial carcinoma cells. EGF might overactivate and cause the functional redundancy of this pathway. These responses are deleterious to UTUC cell proliferation. Alterations in the RTK/RAS/PI(3)K pathway were mutually exclusive in urothelial carcinomas.^[^ ^29^ ^]^ The co‐occurrence *EGFR* and *KRAS* mutations in lung adenocarcinoma causes cell death.^[^ ^30^ ^]^ Hence, molecular targeting therapy against EGFR may not be an efficacious UTUC treatment.

Overgrowth of normal tissue‐derived organoids is a major obstacle in culturing tumor organoids from clinical samples.^[^ ^31^ ^]^ Here, the UTUC organoid cultures were not contaminated by normal organoids because EGF was excluded from the culture medium. EGF is vital to the growth of normal organoids.

The UTUC organoid lines established in the present study recapitulated the features of their corresponding parental tumors in terms of histological architecture, genetic mutations, and biomarker and global gene expression profile. Certain UTUC organoid lines changed their basal or luminal subtypes during culture, which is not surprising and consistent with a previous study on the culture of bladder cancer organoids.^[^ ^15^ ^]^ Its cause is unknown, but the absence of the tumor microenvironment (TME) in the organoid cultures might partially account for it. The prostate cancer organoid line MSK‐PCa2 was negative for pan‐cytokeratin but showed positive staining of this antigen when grown as xenografts.^[^ ^32^ ^]^ In culture, bladder cancer organoid lines lost luminal and gained basal marker expression. When they were grafted into mice, they often reverted to the luminal phenotype of their parental tumors.^[^ ^15^ ^]^ Another study showed that 3D printing of cancer‐associated fibroblasts into bladder cancer assembloids prevented luminal‐to‐basal drift in tumor subtype.^[^ ^33^ ^]^ Hence, a potential solution is to integrate tumor‐associated stromal components into tumor organoid culture systems.

UTUC‐4_O markedly differed from its parental tumor both genetically and transcriptomically perhaps because of imperfect sampling. Organoid cultures prepared from various tumor regions harbored different driver gene mutations and exhibited differential drug responses.^[^ ^16^, ^34^ ^]^ A potential workaround is to derive tumor organoids from whole‐tumor homogenate rather than resected pieces or specific tumor regions. Discrepancies between tumor organoids and their tumor sources may also occur as a result of selecting subclones during in vitro organoid culture. A dominant subclone of biopsy samples appeared in 50% of all colorectal cancer organoid lines.^[^ ^11^ ^]^ Bladder cancer organoid cultures also underwent clonal evolution during serial passaging.^[^ ^15^ ^]^ Therefore, elucidation of the mechanism underlying tumor heterogeneity and evolution may greatly advance the development of organoid culture systems that minimize genetic drift and clonal selection.

Urothelial cancers are chemosensitive and neoadjuvant or adjuvant chemotherapy is recommended for high‐risk UTUC treatment. Chemotherapy might kill residual UTUC cells at the resection site, destroy circulating tumor cells, and reduces tumor recurrence.^[^ ^34^ ^]^ The latter is common among patients with UTUC (22–47% bladder and 2–6% contralateral upper urinary tract recurrence).^[^ ^35^ ^]^ However, chemotherapy outcomes vary widely among individuals as there is extensive intertumoral heterogeneity. We evaluated the feasibility of UTUC organoids as a preclinical model for personalized medicine development by performing high‐throughput drug screenings on them. As the UTUC organoid lines were highly heterogeneous, they dramatically differed in terms of their responses to various compounds. Hence, appropriate chemotherapy regimens must be selected.

Numerous clinical and fundamental studies have been conducted over the past two decades to clarify anticancer drug resistance mechanisms. The output of this research has revolutionized our understanding of cancer cell responses to drug treatment. Nevertheless, it has not yet been confirmed whether drug resistance is driven by pre‐existing subclones or by transcriptional tumor cell reprogramming. Here, we identified various subclones in UTUC tissues and organoids (Figure S2A–J, Supporting information). However, no subclonal evolution was evident in UTUC organoids treated with GEM. Mutations were detected in both untreated and GEM‐treated UTUC organoids (Figure S15, Supporting information). Thus, nongenetic GEM resistance in UTUC organoids may occur through adaptive epigenetic/transcriptional reprogramming. The scRNA‐seq data revealed the pre‐existence of GEM‐tolerant tumor cells, which transcriptionally differed from GEM‐sensitive tumor cells and expanded after GEM treatment. RNA velocity analysis also showed those cells were the transition root (Figure S11, Supporting information). Moreover, this study also revealed that UTUC organoids from two patients displayed distinct responses to gemcitabine treatment, which may explain different responses to this drug among UTUC patients. For these reasons, the responses to gemcitabine varied among patients with UTUC.

Understanding the molecular mechanisms of adaptive drug resistance will aid in the development of efficacious and rational combination therapy strategies. Our scRNA‐seq analysis revealed that c‐Met expression was increased after gemcitabine treatment and the c‐Met inhibitor cabozantinib was synergistically cytotoxic with gemcitabine to UTUC organoids. Cabozantinib might have overcome gemcitabine resistance in UTUC organoids, which is consistent with a previous study in which cabozantinib increased the efficacy of gemcitabine in treatment of pancreatic cancer cells.^[^ ^36^ ^]^ A phase I trial on a combination of cabozantinib and gemcitabine was conducted on advanced pancreatic cancer, but was terminated because of dose‐limiting toxicity.^[^ ^37^ ^]^ Future studies on cabozantinib‐gemcitabine combinations should focus on improving the dosing schedule and reducing toxicity. To be noted, these findings were observed through in vitro experiments conducted on organoids, and no in vivo experiments were performed to validate these findings in the present study. The use of c‐Met as a potential therapeutic target for overcoming gemcitabine resistance should be further tested in future studies.

The development of efficacious chemotherapeutic strategies for UTUCs has been hampered by a lack of clinical and pathological prognostic factors that stratify patients and facilitate clinical decision‐making. Several predictive and prognostic tools have been developed to assess the efficacy of various treatment options.^[^ ^3^ ^]^ Nevertheless, their predictive accuracy remains low. UTUC organoid‐based high‐throughput drug screening could assist in the development of personalized medicine as well as algorithms that accurately predict drug efficacy. In the present study, this process was constrained by the small sample size. The collection of larger numbers of UTUC organoids and the performance of high‐throughput drug testing would increase the statistical power required to detect molecular drug response markers. The correlation of WES and RNA‐seq data with UTUC organoid drug responses will help clarify the mechanism of UTUC carcinogenesis.

Patient‐derived gastrointestinal and gastric cancer organoids emulated clinical drug responses in patients.^[^ ^16^, ^39^ ^]^ Co‐clinical trials should be performed to determine whether the in vitro responses of patient‐derived UTUC organoids to drugs effectively reflect the clinical responses of patients to these chemotherapeutic agents.

## 4 Experimental Section

### Sample Collection

Fresh UTUC samples, normal tissues adjacent to the tumors, and matched blood samples were obtained from patients in the Shenzhen Second People's Hospital. All patients provided informed consent and this study was conducted according to the guidelines of the local research ethics committees (Research Ethics Committee of Shenzhen Second People's Hospital, No. 20210219002) and local law. Please see Table S1, Supporting information for the clinical details of these samples.

### Organoid Culture

Tumor samples were washed twice with cold PBS and minced into small pieces using scissors. A small random piece was fixed in 10% neutral‐buffered formalin for histopathological analysis and immunofluorescence staining. Two pieces of minced tissue were snap frozen and stored at −80 °C for DNA and RNA isolation, while the rest was used for the isolation of cancer cells. The blood samples were aliquoted at 500 µL/tube, snap frozen and stored at −80 °C for DNA isolation. The tissues for the derivation of organoids were further minced into <2 mm pieces in a 10 cm tissue culture plate and dissociated using collagenase II (5 mg mL^−1^) with ROCK inhibitor Y‐27632 dihydrochloride (10 × 10^−6^ m) for 1 h at 37 °C. Digested tissues were spun down at 200 g for 5 min, washed once with AdDMEM/F12. The tissues were then incubated in 5 mL of TrypLE Express with Y‐27632 dihydrochloride (10 × 10^−6^ m) for 5 min at 37 °C. Digestion was stopped by the addition of 10 mL of AdDMEM/F12 supplemented with 1% penicillin/streptomycin, 1% Glutamax, 1% HEPES (Gibco), and 20% FBS. After centrifugation, 2 mL of AdDMEM/F12 supplemented with 20% FBS was added and the solution was pipetted several times to further dissociate the tissues. The dissociated cells were then filtered through a 70 µm cell strainer to remove large, undigested clusters. The pellet after centrifugation was resuspended with cold organoid medium and mixed with cold Matrigel. 30 µL droplets of Matrigel‐cell mixture (≈20 000 cells per drop) were deposited into 6‐well plates. The cell culture plate was turned upside down and the Matrigel drops were solidified for 10 min at 37 °C and 5% CO_2_. Upon Matrigel solidification, 2.5 mL of UTUC organoid medium was added to each well. See Table S2, Supporting information for the composition of the UTUC organoid medium. The medium was replaced every 2–3 d. Y27632 was withdrawn from the medium after 7 d.

For passaging, Matrigel drops were scraped from the plate and centrifuged to remove the culture medium. The Matrigel surrounded the organoids was removed by incubating with 2 mL of TrypLE for 5 min at 37 °C. The trypsinization was stopped by the addition of 6 mL of AdDMEM/F12 supplemented with 20% FBS. After centrifugation, organoids were pipetted up and down for several times. Dissociated organoids were centrifuged to remove the medium and seeded as above mentioned. Organoids were passaged at a 1:2–1:3 ratio every 2–3 weeks.

To cryo‐preserve organoids, organoids were dissociated from the Matrigel, dissociated into small clusters and then frozen in recovery‐cell‐culture freezing medium (GIBCO).

### Histology and Immunostaining

Tissues and UTUC organoids were washed in PBS and fixed in 10% neutral buffered formalin. The samples were then subjected to dehydration, embedding and sectioning. H&E staining and immunostaining were performed on 4 µm paraffin sections. For H&E staining, paraffin sections were deparaffinized in xylene and rehydrated through a graded‐ethanol series. For immunofluorescence experiments, paraffin slides were subjected to antigen retrieval by incubation in boiling EDTA solution (pH 8.0) after being deparaffinized and rehydrated. Sections were blocked in 5% BSA in PBS for 1 h at 37 °C and incubated with diluted primary antibodies (listed in Table S3, Supporting information) in 3% BSA in PBS overnight at 4 °C. Slides were then washed 3 times with PBS and incubated with diluted secondary antibodies (listed in Table S3, Supporting information) in 3% BSA in PBS for 1 h at room temperature. Nuclei were counterstained with DAPI. Immunofluorescence was imaged using a ZEISS confocal microscope.

### WES Analysis

The genomic DNA was isolated from cancer tissues, blood, and cultured organoids using the AllPrep DNA/RNA Mini Kit (Qiagen). WES libraries were generated using Agilent SureSelect Human All Exon V6 kit (Agilent Technologies, CA, USA) according to the manufacturer's instructions. Index codes were added to each sample. The exome was sequenced with paired‐end (2 × 150 bp) runs using Illumina NovaSeq. The blood samples were sequenced with sequencing depths of 100 × (≈12 Gb per sample) and tumor and organoids samples with depths of 200 × (≈24 Gb per sample). Fastp (v0.12.6) was used to remove adaptors and filter low quality reads.^[^ ^39^ ^]^ Single‐nucleotide variant (SNVs) were called by GATK(v4.1.9).^[^ ^40^ ^]^ Sequence reads were mapped against the human reference genome (hg38) using Burrows‐Wheeler Alignment with maximal exact matches (BWA‐MEM) v0.7.12.^[^ ^41^ ^]^ Read mapping was followed by the marking of duplicates, merging of lanes, and realignment of indels using Picard (v2.23.8). Somatic SNVs and indels in the tumors and organoids were called with Mutect2 (default options) using the matched blood samples as the reference. Somatic SNVs with a VAF < 0.05 and supported by less than 3 reads were filtered out. Mutation effect predictions and annotations were performed by VEP (release 101).^[^ ^42^ ^]^ CNVs were detected using TitanCNA (1.30.0).^[^ ^43^ ^]^ The clone architecture and the VAF of mutated sites in organoids and the corresponding tumors were inferred by sciClone.^[^ ^44^ ^]^

### RNA‐seq and Analysis

RNA was isolated from cancer tissues and cultured organoids using the AllPrep DNA/RNA Mini Kit (Qiagen). Sequencing libraries were generated using NEB Next UltraTM RNA Library Prep Kit for Illumina (NEB, USA) following the manufacturer's instructions. Index codes were added to attribute sequences to each sample. The RNA libraries were sequenced with paired‐end 150 bp using Illumina NovaSeq. Low‐quality and adaptor polluted reads were removed. Then sequenced reads were aligned to the reference genome (Ensembl hg38) using STAR(v2.4.0j).^[^ ^45^ ^]^ Gene expression analysis was performed by RSEM.^[^ ^46^ ^]^ The molecular subtypes of UTUC organoids and tumor tissues were identified using BLCAsubtyping and consensusMIBC in R.^[^ ^47^ ^]^ For BLCAsubtyping script, the built‐in classification signatures of UNC (BASE47)^[^ ^25^ ^]^ and TCGA^[^ ^48^ ^]^ were used. Top 30% variably expressed genes were used for PCA plot and Euclidean distance calculation (Figure 4B,D). The pathway enrichment scores were calculated by gsva method using GSVA package in R.^[^ ^49^ ^]^ Gene sets were downloaded from the GSEA official website.

### Organoid Drug Screening

UTUC organoids were dissociated into small clusters, filtered through a 100 µm cell strainer to remove large organoids and plated in low‐attachment 96‐well plates in 2% Matrigel/growth medium (20 000 organoids mL^−1^) in two technical replicates. A six‐point fivefold dilution series of 24 drugs were dispensed 24 h after plating. The maximal concentration of each drug can be found in Table S4, Supporting information. Cell viability was measured using Cell Titer‐Glo 3D (Promega) according to the manufacturer's instructions after 6 d of drug incubation. Screens were performed in technical duplicates and biological triplicates.

### scRNA‐seq and Data Analysis

Organoids from UTUC_1 and UTUC_10 before and after treatment with gemcitabine (0.1 × 10^−6^ m) for 6 d were collected and then gently dissociated into single‐cell suspension followed the manufacturer's guidance. Cell viability was quantified using the trypan blue exclusion method, dead cells were removed using a Dead Cell Removal Kit (Miltenyi Biotec) and cell concentration was adjusted for 10 000 cells per sample using the 10X Genomics Single Cell 30 Reagent Kits v2 protocol to prepare libraries. Libraries were sequenced on the NovaSeq 6000 platform. BCL files were then de‐multiplexed with the 10X Genomics i7 index using Illumina's bcl2fastq and mkfastq command from 10X Genomics CellRanger v4.0.0 tools. Extracted paired‐end fastq files were mapped to the genome (hg38) and the raw expression matrix for each sample was generated using cellRanger count function. The raw UMI (unique molecular identifier) count matrix for each sample was processed using Seurat3 script in R.^[^ ^50^ ^]^ Doublet cells were detected using DoubletFinder with default parameters.^[^ ^51^ ^]^ Cells with nFeature_RNA > 10 000 or < 2000, or nCount_RNA > 75 000, or with more than 25% mitochondrical‐derived UMI counts were considered low‐quality cells. Low‐quality cells and doublet cells were rejected for later analysis. Finally, 26 368 cells were remained, and used for downstream analysis. After quality control, all samples were merged. The classic Seurat analysis pipeline was used. Briefly, the UMI matrix was log2 normalized and the most 3500 variable genes were selected. Then, the dimension reduction was processed using PCA with top 20 PCs followed by UMAP and tSNE. Finally, the cells were clustered using the K‐nearest neighbors graph‐based method. Single‐cell level GSEA was performed using VAM (Variance‐adjusted Mahalanobis).^[^ ^52^ ^]^ Differential expression analysis and differential pathway enrichment analysis were analyzed based on Wilcoxon rank sum test using presto script (https://github.com/immunogenomics/presto). The cell cycle analysis was performed using cyclone script in scran package.^[^ ^53^ ^]^ The RNA velocity were analyzed using scVelo software.^[^ ^26^ ^]^

### Creation of Gene Signatures

Two studies (GSE116118 and GSE106336) were used for generating the gemcitabine resistance signature.^[^ ^54^ ^]^ For each data, limma^[^ ^55^ ^]^ package was used for differential expression analysis, and genes with *p*‐value < 0.01 and log_2_(Fold Change, FC) > 2 in gemcitabine resistant cell lines were used as gemcitabine resistance signature (These two signatures were labeled as GSE116118.GEM and GSE106336.GEM in the manuscript). The significantly upregulated or downregulated genes with log_2_FC > 0.25 and adjust *p*‐value < 0.01 in C4, C5, C8, and C9 were used as gemcitabine resistance cluster specific upregulated signature and downregulated signature (We labeled them as C4.hi.signature, C4.lo.signature, C5.hi.signature, C5.lo.signature, C8.hi.signature, C8.lo.signature, C9.hi.signature, C9.lo.signature). GSEA analysis (Figure 6E and Figure S10, Supporting information) and universal enrichment analysis (Figure S12, Supporting information) were performed using clusterProfiler package.^[^ ^56^ ^]^

### qPCR

Total RNA was extracted from organoids using RNeasy Plus Mini Kit (Qiagen). cDNA was generated from 1 µg of total RNA using ReverTra Ace qPCR RT Master Mix (Toyobo). qPCR was performed with QuantStudio 3 (Applied Biosystems, NY) using SYBR Green Realtime PCR Master Mix (Toyobo). The primer sequences were listed in Table S5 (Supporting information). The mRNA levels of target genes were normalized to the expression of the housekeeping gene *β‐actin*.

### Statistical Analysis

Sample size (*n*) for each statistical analysis is provided in the relevant figure legends. The analysis of sequence data was described in the Experimental Section. The differences in Euclidean distances between groups in Figure 4D were analyzed using the top 30% variably expressed genes. The IC_50_ values and AUC shown in Figure 5 and Figure S7 (Supporting Information) were calculated using GraphPad Prism 7 by applying nonlinear regression (curve fit) on the equation log (inhibitor) versus response (variable slope). The differences in logIC_50_ values of trametinib on *FGFR3* mutated and wildtype UTUC organoid lines shown in Figure 5D were analyzed using Wilcoxon test. Differential enrichment analysis of signaling pathways shown in Figure 6F was performed using Wilcoxon rank sum test. The differences in gene expression shown in Figure 6G and Figure S14 were analyzed using GraphPad Prism 7 by applying unpaired two‐tailed *t*‐test. *p < 0.05; **p < 0.01; ***p < 0.001; ns, not significant.

## Conflict of Interest

The authors declare no conflict of interest.

## Author Contributions

Z.L., H.X., Y.G., W.C., and Y.Z. contributed equally to this work, conducted experiments, and wrote the manuscript. L.Y., Y.S., A.L., S.H., B.G., Y.W., G.X., D.F., Y.H., Q.T., L.Y., and H.M. prepared and processed the clinical samples. Z.H., Z.H., Z.C., Y.G., X.L., L.Z., and W.H. conceived the project. W.H. supervised the process of the study and revised the manuscript.

## Supporting information

Supporting Information

## Data Availability

The data that support the findings of this study are available from the corresponding author upon reasonable request.
