# Supplementary material for: Patient‐Derived Upper Tract Urothelial Carcinoma Organoids as a Platform for Drug Screening
Source: Adv Sci (Weinh). 2021 Dec 16;9(4):2103999. doi: 10.1002/advs.202103999 (PMC8811809; doi:10.1002/advs.202103999)
Supplement: Supplementary file 1 — Supporting Information [file ADVS-9-2103999-s001.pdf]

ADVANCED SCIENCE
Open Access

Supporting Information

for *Adv. Sci.*, DOI: 10.1002/advs.202103999

Patient-derived Upper Tract Urothelial Carcinoma Organoids as a Platform for Drug Screening

Zhichao Li, Haibo Xu, Yanqing Gong, Wei Chen, Yonghao Zhan, Lei Yu, Yangyang Sun, Aolin Li, Shiming He, Bao Guan, Yucai Wu, Gengyan Xiong, Dong Fang, Yuhui He, Qi Tang, Lin Yao, Zheng Hu, Hongbing Mei, Zhisong He, Zhiming Cai, Yinglu Guo, Xuesong Li, Liqun Zhou*, and Weiren Huang**

Supporting Information

for *Adv. Sci.*, DOI: 10.1002/advs.202103999

Patient-derived Upper Tract Urothelial Carcinoma Organoids as a Platform for Drug Screening

Zhichao Li, Haibo Xu, Yanqing Gong, Wei Chen, Yonghao Zhan, Lei Yu, Yangyang Sun, Aolin Li, Shiming He, Bao Guan, Yucai Wu, Gengyan Xiong, Dong Fang, Yuhui He, Qi Tang, Lin Yao, Zheng Hu, Hongbing Mei, Zhisong He, Zhiming Cai, Yinglu Guo, Xuesong Li, Liqun Zhou*, and Weiren Huang**

Supporting Information

Patient-derived Upper Tract Urothelial Carcinoma Organoids as a Platform for Drug Screening

Zhichao Li, Haibo Xu, Yanqing Gong, Wei Chen, Yonghao Zhan, Lei Yu, Yangyang Sun, Aolin Li, Shiming He, Bao Guan, Yucai Wu, Gengyan Xiong, Dong Fang, Yuhui He, Qi Tang, Lin Yao, Zheng Hu, Hongbing Mei, Zhisong He, Zhiming Cai, Yinglu Guo, Xuesong Li, Liqun Zhou*, and Weiren Huang**

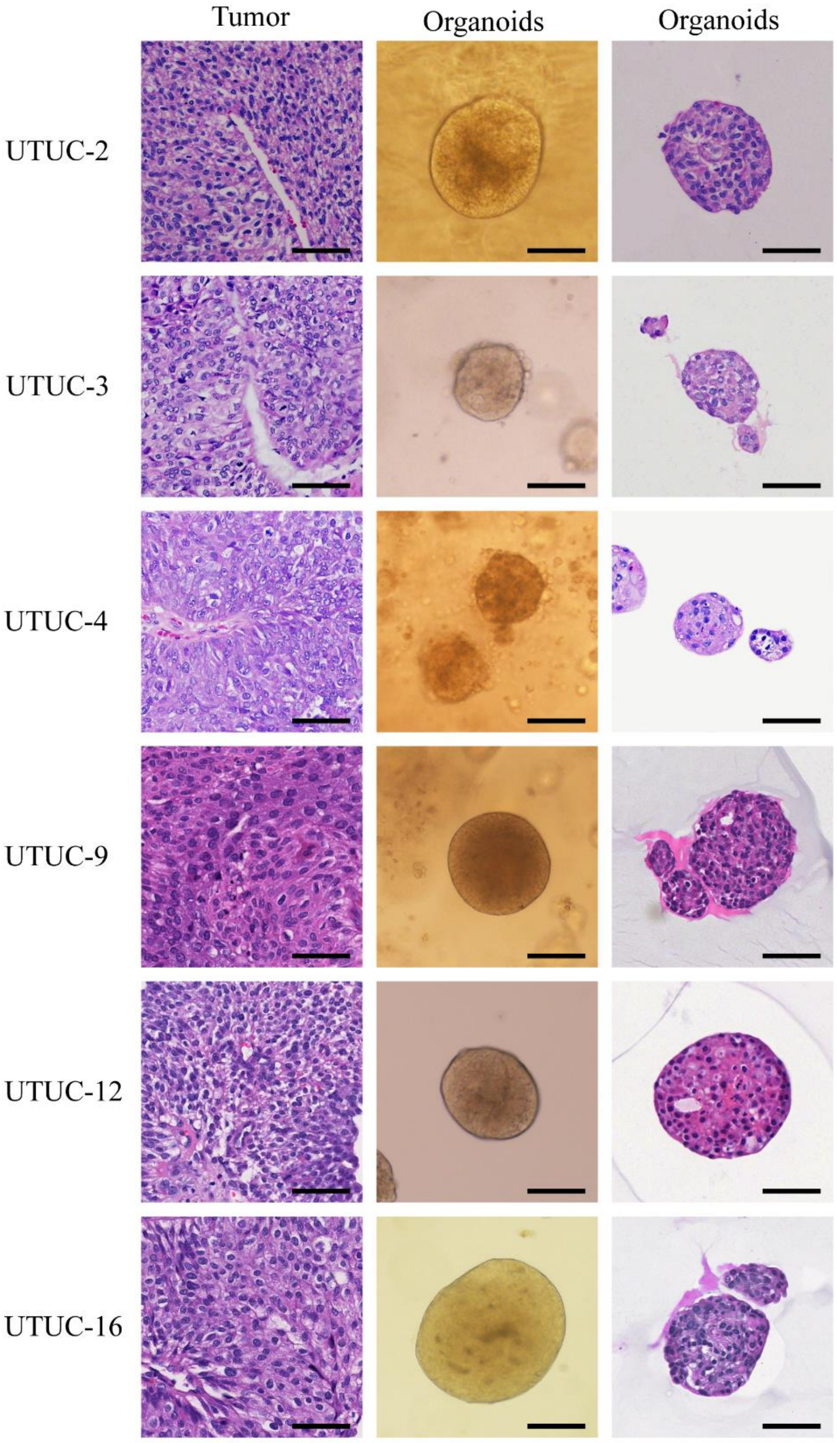

Figure S1. Representative H&E staining images and bright-field images of organoids and corresponding parental tissues. Scale bar, 100 μm.

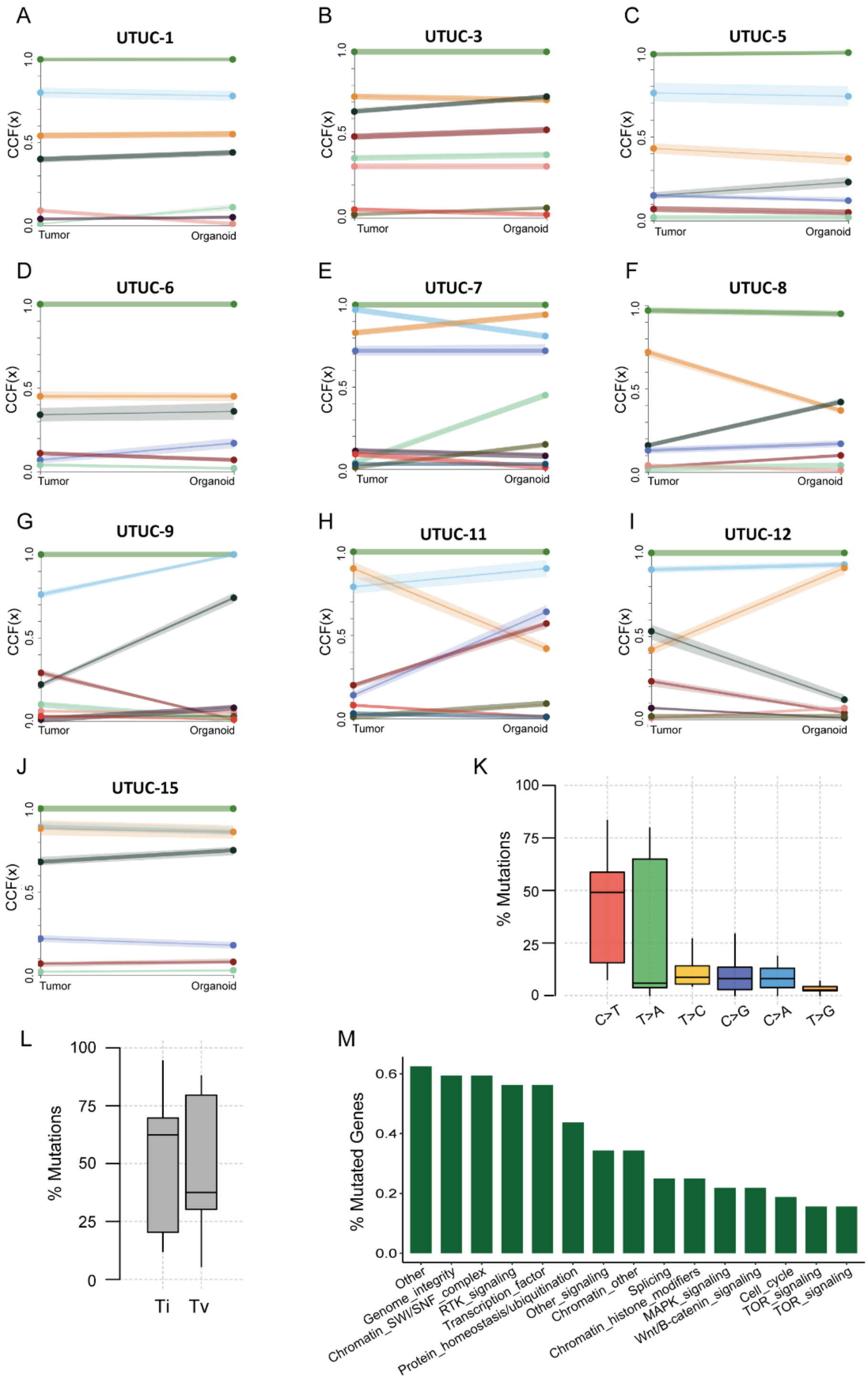

Figure S2. Mutational evolution and clonal alteration in UTUC organoids. A–J) Line plots of mutant clones and cancer cell fraction in UTUC organoids and tumor tissues. Each dot with a different color represented a mutant clone identified by PhylogicNDT in either tumor tissue (left) or organoid (right). Y axis showed the CCF value of each specific clone calculated by PhylogicNDT. CCF value represented the fraction of cancer cells contained the corresponding clone mutations. The lines connected the same mutant clones (same color) showed the CCF variation tendency between tumor tissues and organoids. The green line (near 1 for many samples) connected the main mutant clones in both tumor tissues and organoids. K and L) Percentage of the six types of base substitutions (K) and the transitions and transversions spectrum (L) across all samples. Graphs shown are mean ± SD. M) The enriched pathways of mutated genes in UTUC samples.

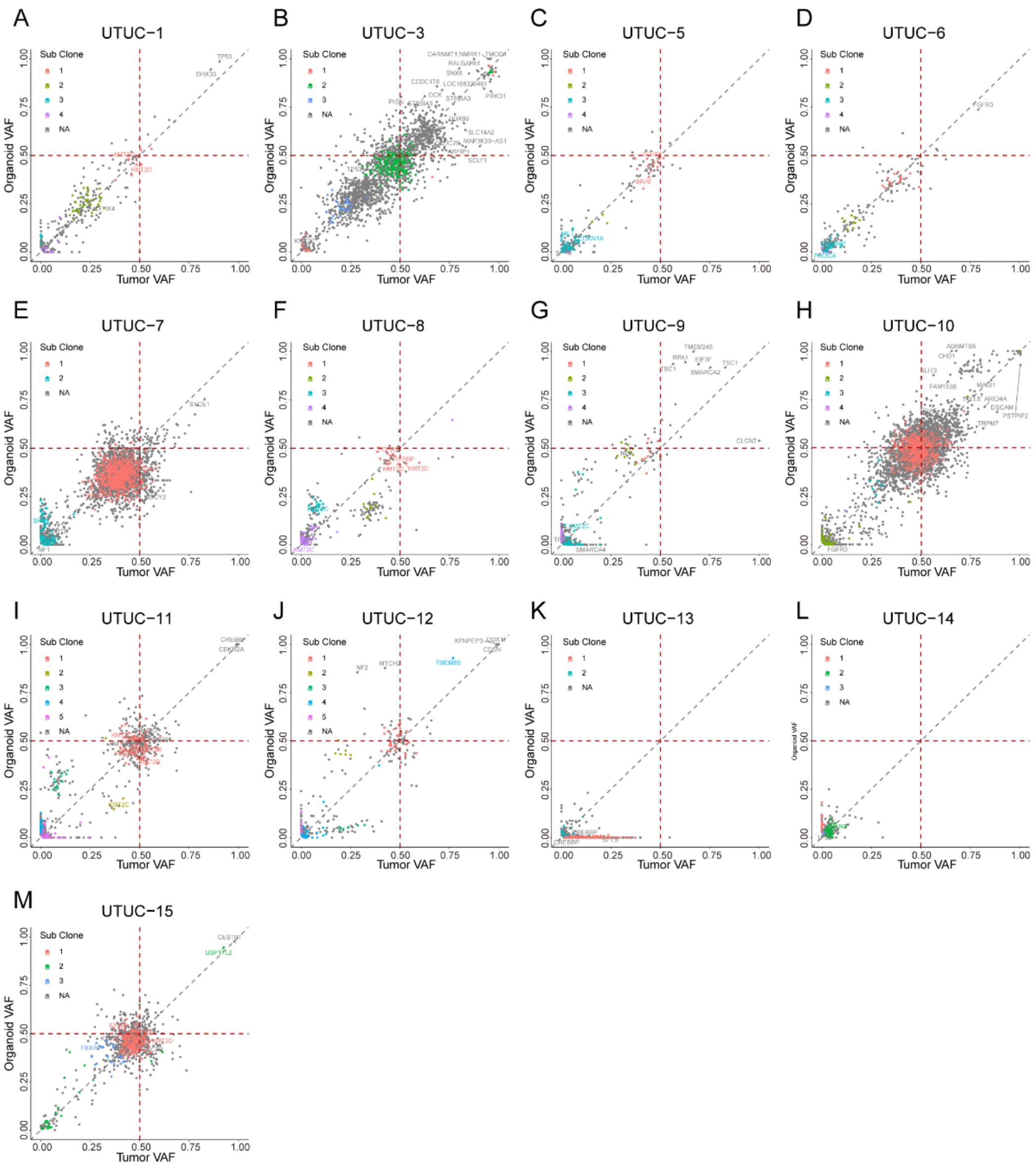

Figure S3. Subclonal composition of UTUC tissues and organoids. A–M) Dot plots showing the VAF of mutated sites in each tumor and the corresponding organoid line. Different colors showed the subclones identified in each sample using sciClone script. Genes listed in the figures were those with a VAF value > 0.8 or used in Figure 3A.

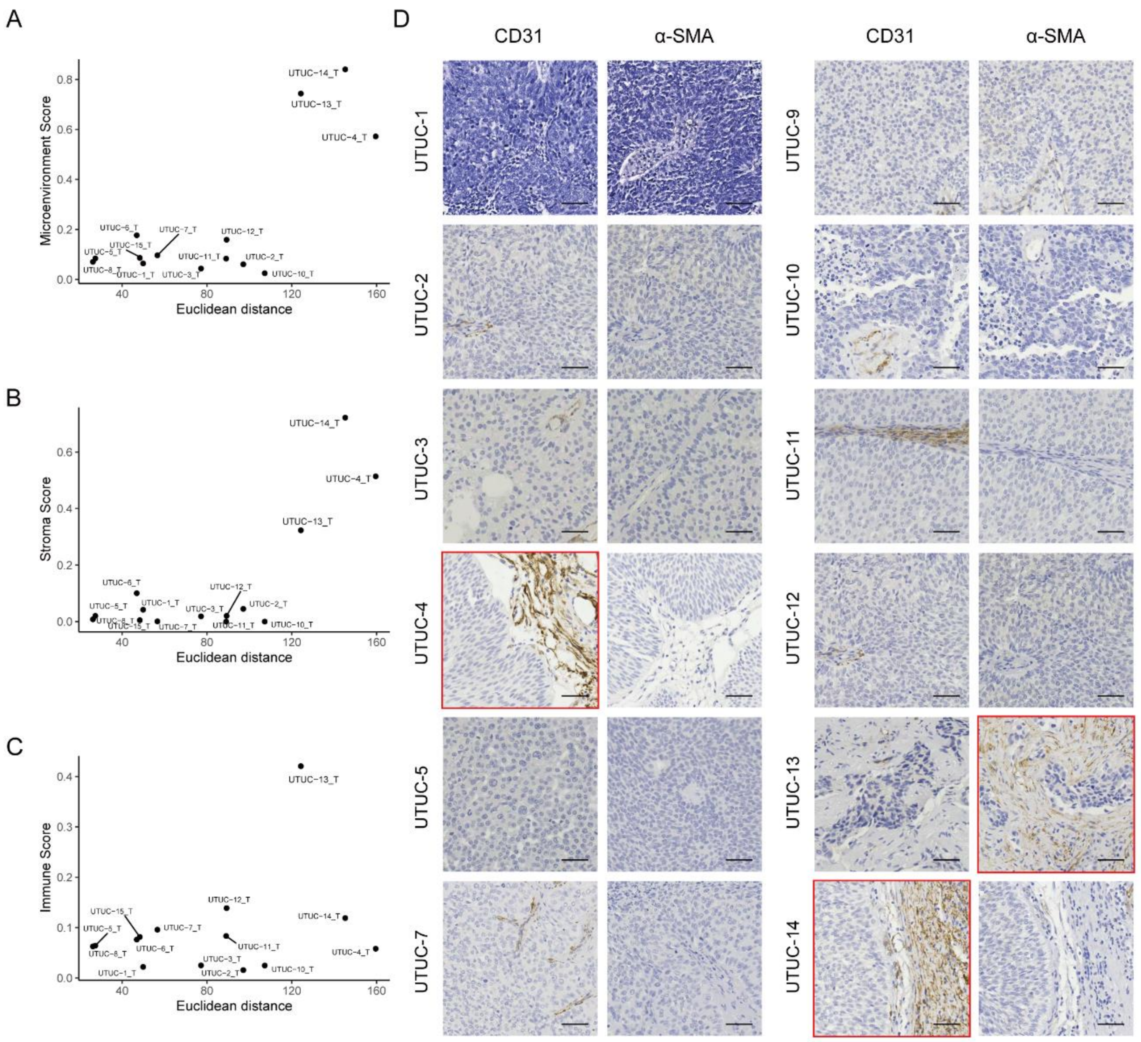

Figure S4. Analysis of the stromal components in UTUC tumor tissues. A–C) Dot plots showing the association between the Euclidean distance and microenvironment score (A), stroma score (B) and immune score (C), respectively. These tumor microenvironment (TME) scores were calculated using xCell. D) Representative immunohistochemistry images of UTUC tumors for CD31 and α-SMA. Scale bar, 50 μm.

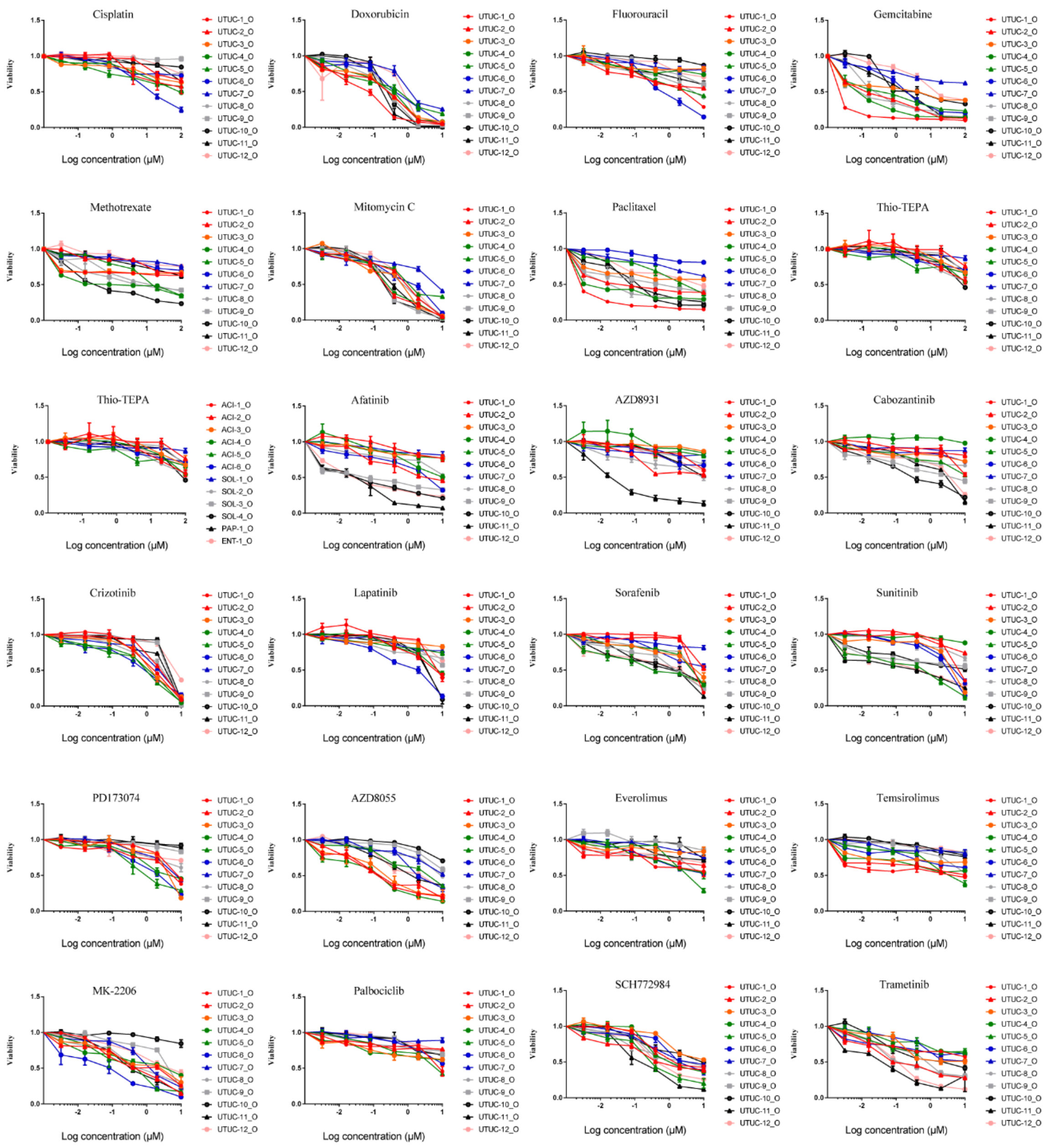

Figure S5. Drug response analyses of UTUC organoids, related to Figure 5. Shown are the dose response curves of 24 compounds against 12 UTUC organoid lines. Each data point represents three biological replicates, with error bars representing ± SEM.

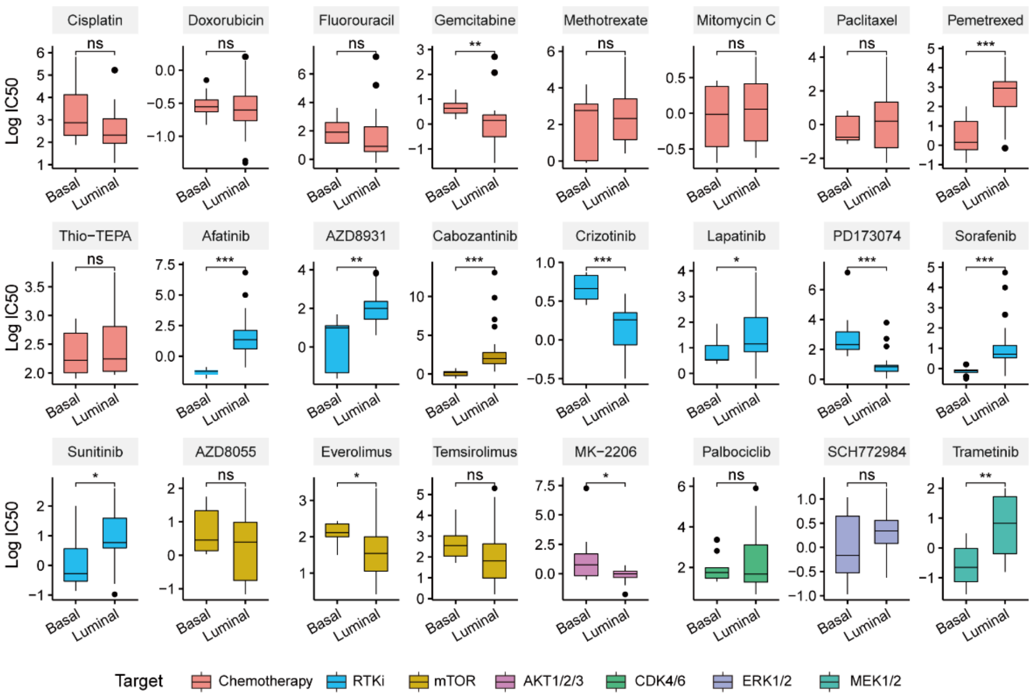

Figure S6. Box plots showing the differences in drug responses between basal and luminal UTUC organoid lines. ns, not significant, *$p < 0.05$, **$p < 0.01$, **$p < 0.001$.

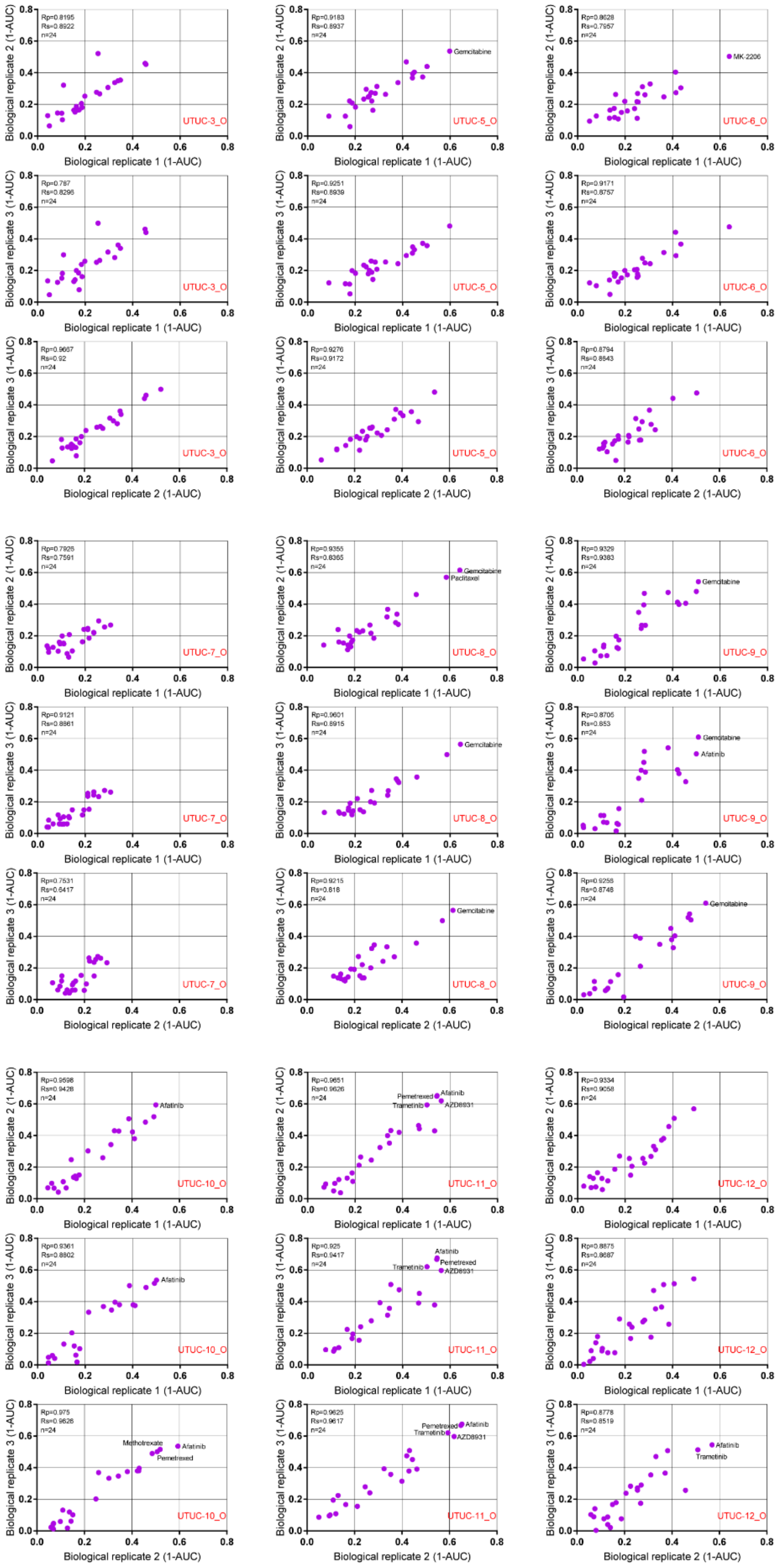

Figure S7. The correlation of 1-AUC values from biological replicates, related to Figure 5. Shown are the scatterplots of 1-AUC values from two biological replicates of the drug screening data, highlighting drugs (red) having an obvious inhibitory effect on viability (1-AUC > 0.5 for both biological replicates) of indicated organoid lines.

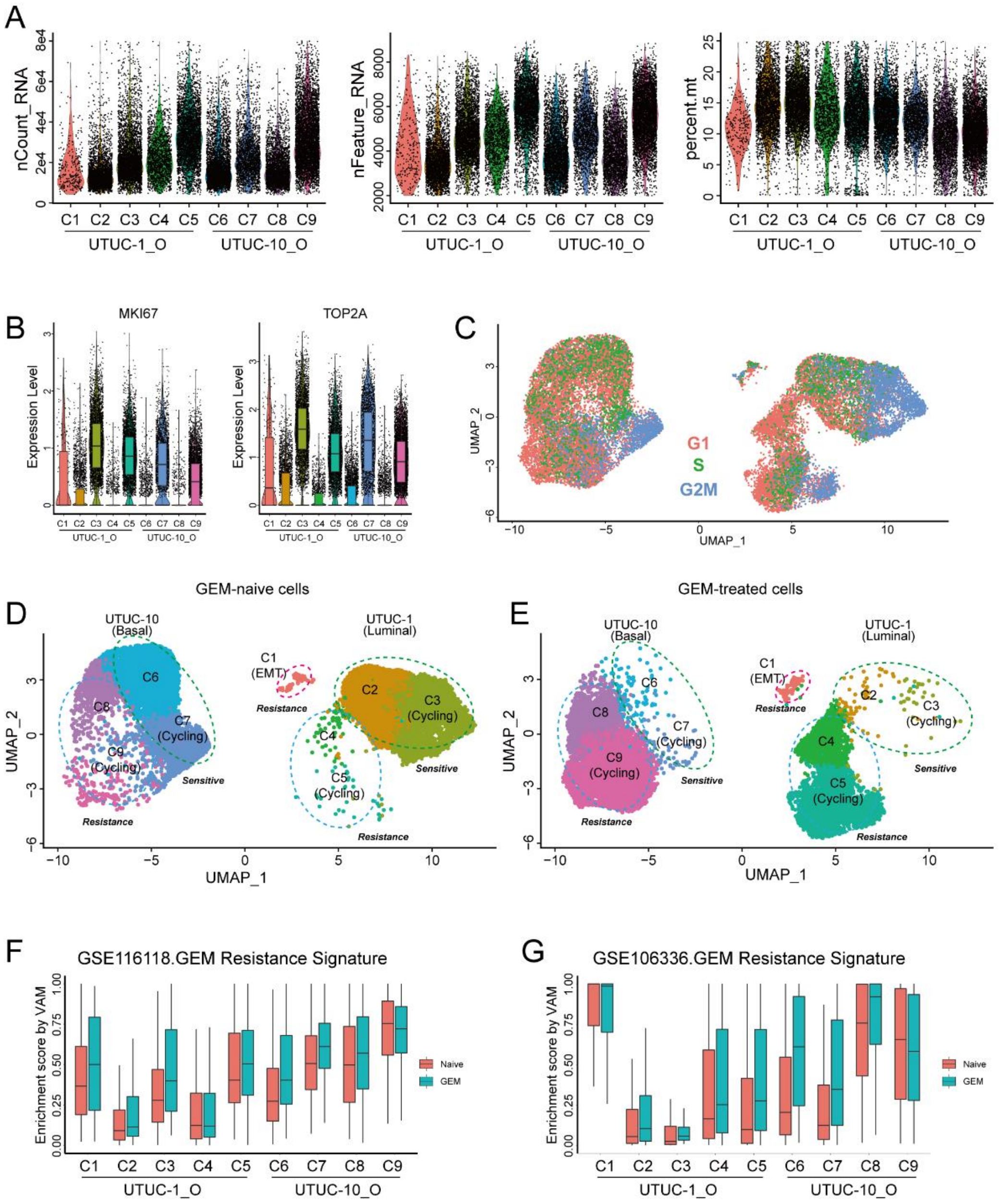

Figure S8. The gene signature and heterogeneity of UTUC organoids. A) Violin plots showing the quality of cells in each cluster based on the value of 'nCount_RNA', 'nFeature_RNA', and 'percent.mt'. B) Boxplots showing the expression levels of MKI67 and TOP2A in each cluster. C) UMAP plot showed the distribution of cells in each cell cycle stage. D, E) UMAP plot showing the distribution of cells before (D) and after (E) GEM treatment. F, G) Boxplot showing the enrichment score of GSE116118.GEM Resistance Signature (F) and GSE106336.GEM Resistance Signature (G) in each sub-cluster before (color in red) and after (color in blue) GEM treatment.

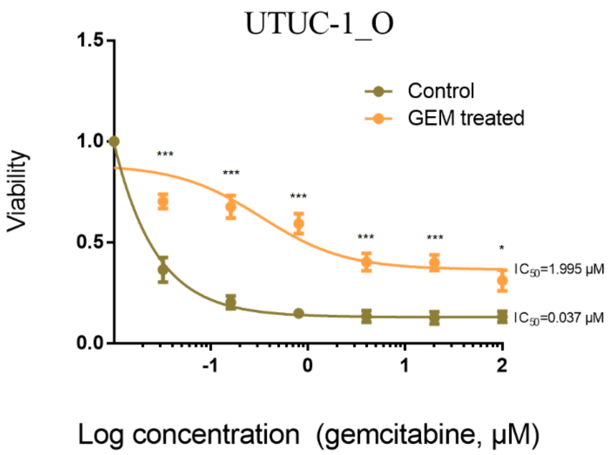

Figure S9. Dose-response curves of control and GEM-treated UTUC-1_O organoids treated with GEM. GEM-treated UTUC-1_O indicated organoids regrown from cells survived one-week GEM (0.1×10^{-6} M) treatment. Each data point represents three biological replicates, with error bars representing ± SEM. *$p < 0.05$, ***$p < 0.001$.

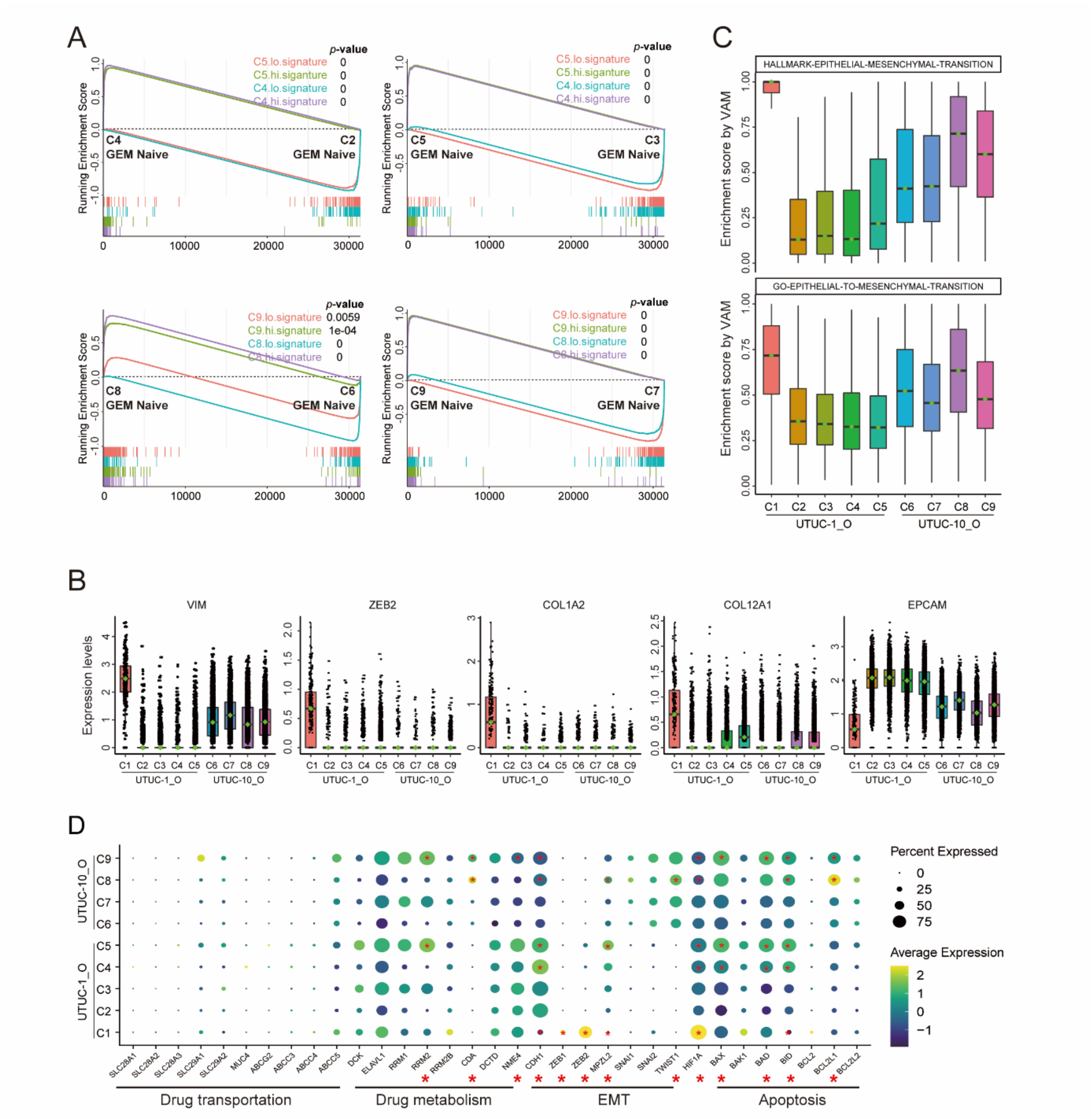

Figure S10. The analysis of GEM-resistance signature and EMT signature in UTUC organoids. A) GSEA plots showing the enrichment state of C4–C5 signature, C8–C9 signature between GEM-naive resistant cluster and GEM-naive sensitive cluster in UTUC-1_O (upper panel) and UTUC-10_O (lower panel), respectively. B) Boxplot showing the expression levels of EMT associated genes in each sub-cluster. C) Boxplot showing the enrichment scores of EMT pathways in each sub-cluster. D) Dot plot showing the expression levels of previously reported GEM resistance-associated genes in each cluster.

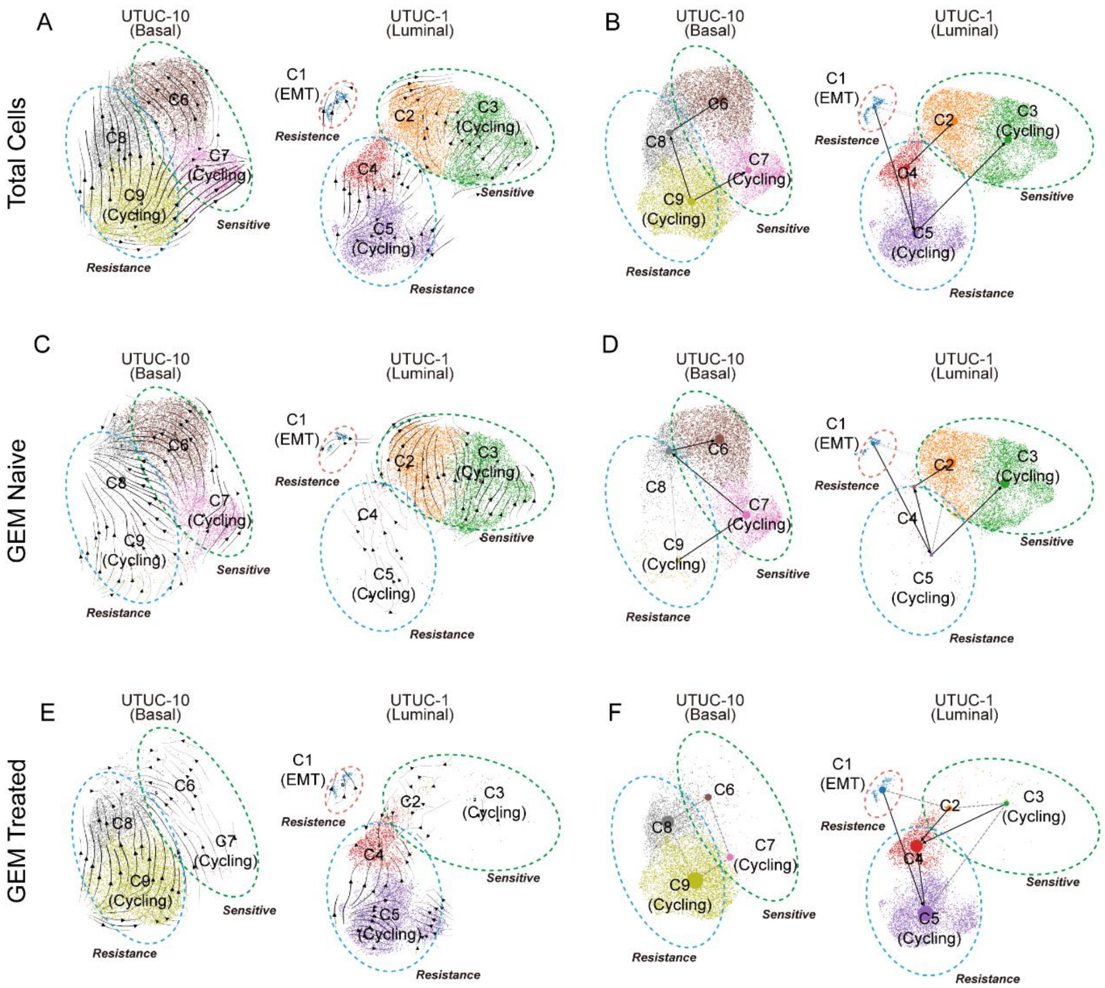

Figure S11. RNA velocity analysis of UTUC organoids. Velocities derived from the dynamical model were visualized as streamlines (A, C, E) or PAGA graph (B, D, F) in a UMAP-based embedding. A, B) The RNA velocity results using scRNA-seq data from all organoids. C, D) The RNA velocity results using scRNA-seq data from GEM-naive organoids. E, F) The RNA velocity results using scRNA-seq data from GEM treated organoids. Streamlines in A, C, E represented the transition trajectory. PAGA graph in B, D, F represented the velocity-inferred direction.

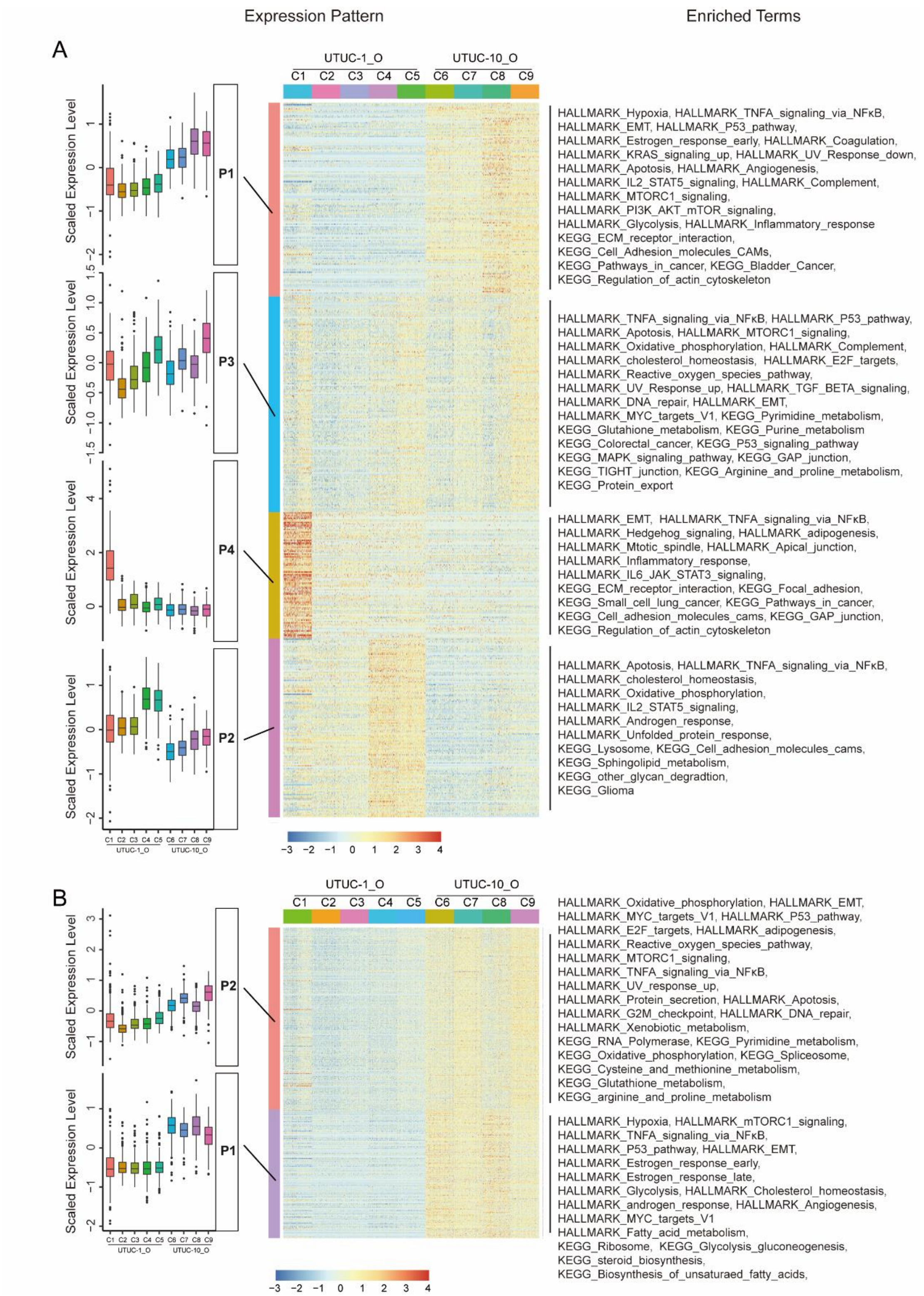

Figure S12. The pattern of differentially expressed genes and enriched pathways in UTUC organoids. A) The expression pattern (left) and enrichment pathways (right) of differential expressed genes (middle) between GEM-naive and GEM-treated UTUC organoids. B) The expression pattern (left) and enrichment pathways (right) of differential expressed genes (middle) between UTUC-1_O and UTUC-10_O before treatment with GEM. A maximal of 200 cells were randomly selected for each cluster to generate the two plots.

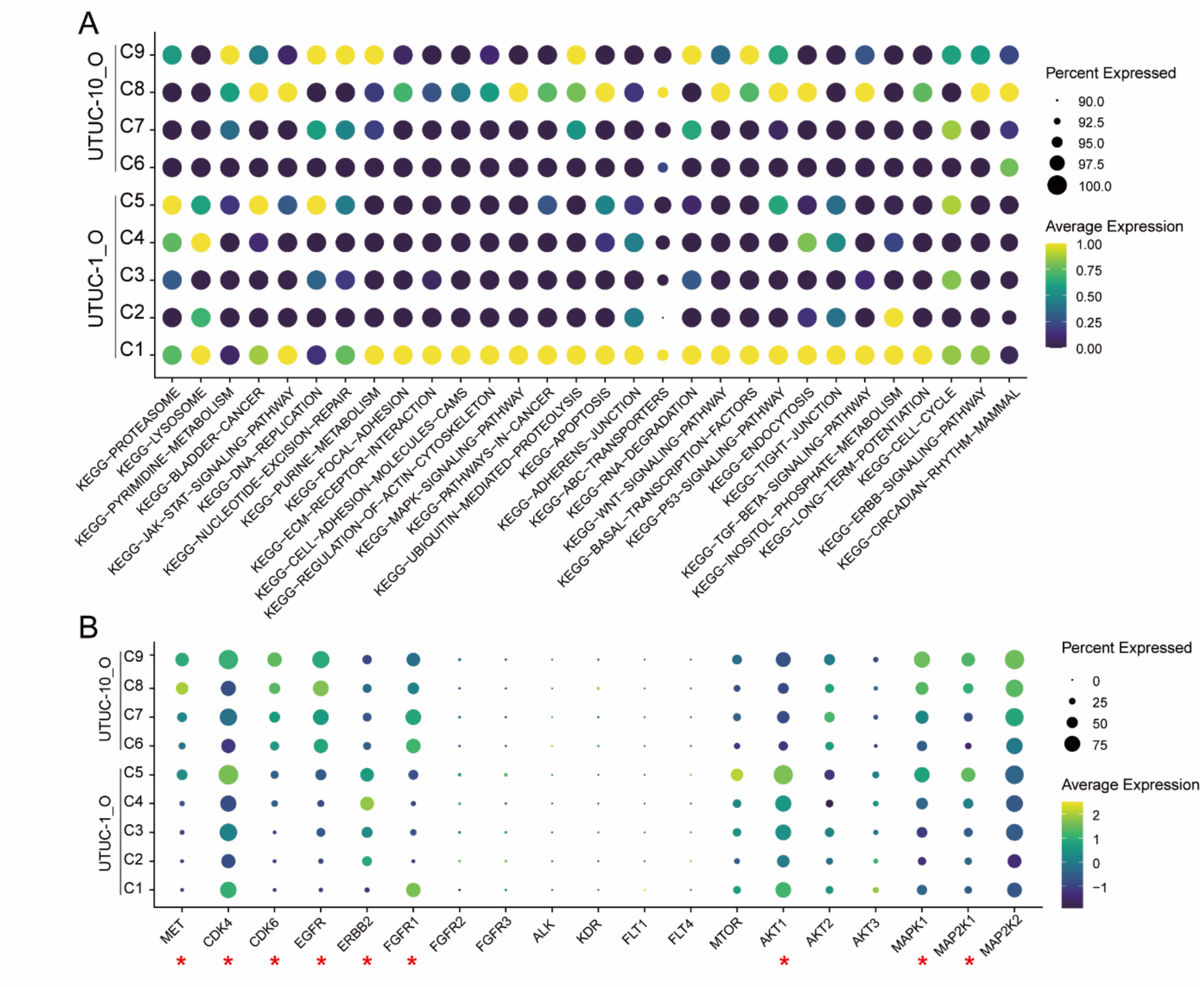

Figure S13. The altered pathways and genes in UTUC organoid after treated with GEM. A) Dot plot showing the KEGG pathways which were highly enriched in GEM-resistant clusters or the EMT cluster. B) Dot plot showing the expression levels of targeted genes in each cluster. Gene labeled with star means the gene was significantly up-regulated in GEM-resistant clusters.

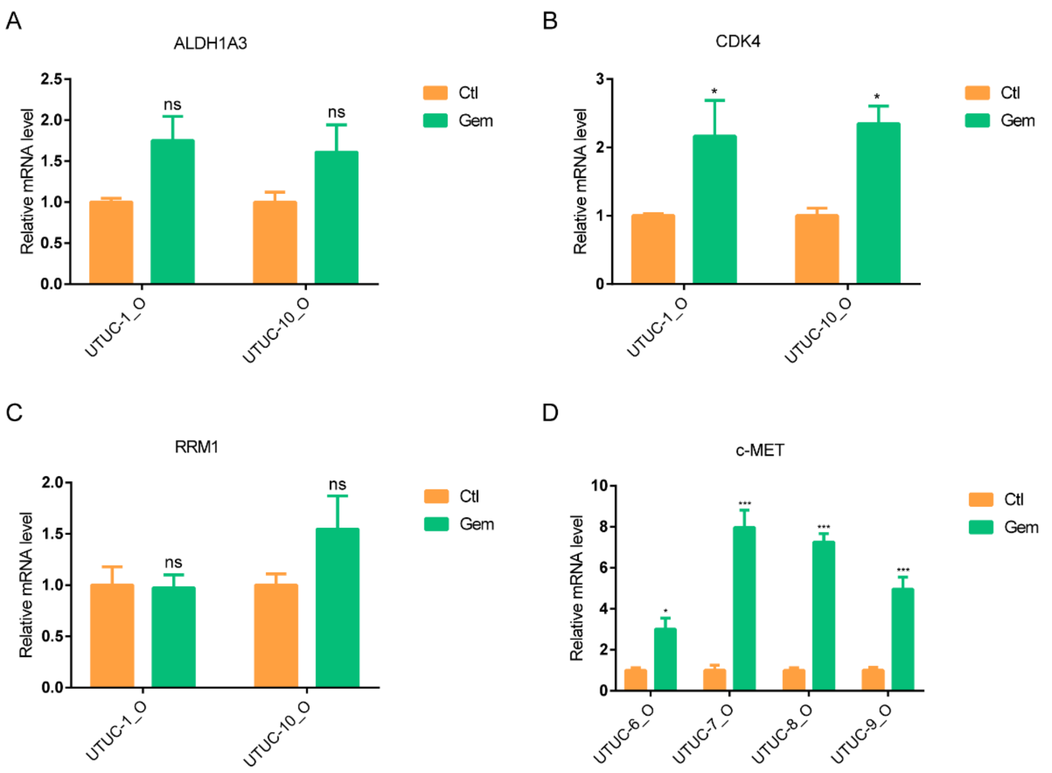

Figure S14. The expression levels of indicated genes in UTUC organoids after treatment with gemcitabine. A–C) QPCR showing the expression level of ALDH1A3, CDK4, and RRM1 in UTUC-1_O and UTUC-10_O after treatment with gemcitabine. D) qPCR showing the expression level of c-MET in UTUC-6_O, UTUC-7_O, UTUC-8_O, and UTUC-9_O after treatment with gemcitabine. Each data point represents three biological replicates, with error bars representing ± SEM. ns, not significant, *$p < 0.05$ and ***$p < 0.001$.

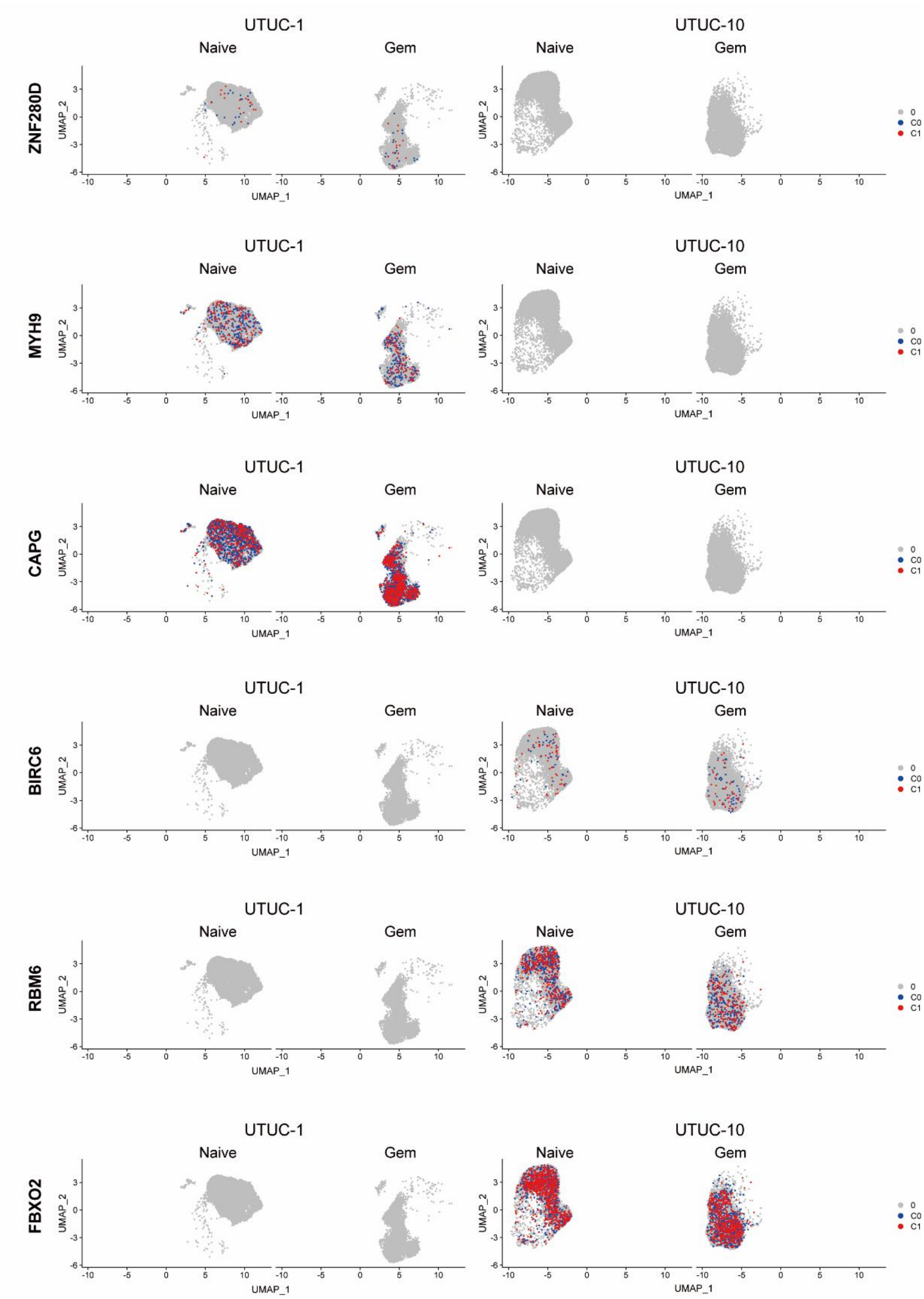

Figure S15. The spectrum of mutations in UTUC organoids after treatment with gemcitabine. The spectrum of mutations, previously identified by WEG in UTUC-1 and UTUC-10, in UTUC organoids after treatment with gemcitabine using 10X Genomics scRNA-seq data. Dots in red or blue meant at least one sequenced read in this cell contained the mutated nucleotides or the reference nucleotides, respectively. Dots in grey meant no reads mapped to the mutated nucleotides sites in the corresponding genes. Here, we show part of the mutated genes in UTUC-1 and UTUC-10.

Table S1. Clinical information of UTUC patients.

Patients' information									
Line	Age	Gender	Location	Grade	pT Stage	Tumor size	History of bladder urothelial carcinoma	Prior therapy	Normal organoid culture
UTUC-1_O	67	F	Renal pelvis	High	II	3.5cm×3cm×3cm	Yes	None	Failed
UTUC-2_O	68	M	Renal pelvis	High	III	1cm×0.5cm×0.5cm	No	None	
UTUC-3_O	80	F	Renal pelvis	Low	I	1.5cm×1cm×0.5cm	Yes	None	Failed
UTUC-4_O	60	M	Ureter	High	III	4.8cm×3.5cm×2.5cm	No	None	Success
UTUC-5_O	74	M	Renal pelvis	High	III	5cm×2.5cm×2cm	No	None	Success
UTUC-6_O	67	M	Ureter	High	I	10.3cm×7cm×1.3cm	No	None	
UTUC-7_O	67	F	Renal pelvis	Low	III	8cm×5cm×3cm	No	None	
UTUC-8_O	56	M	Ureter	Low	Ta	4cm×2.5cm×1cm	Yes	None	Success
UTUC-9_O	64	M	Renal pelvis	High	III	5.5cm×4cm×3cm	No	None	Failed
UTUC-10_O	81	F	Ureter	High	II	4cm×1cm×1cm	No	None	
UTUC-11_O	78	F	Renal pelvis	High	II	4.5cm×3cm×1.5cm	Yes	None	Failed
UTUC-12_O	67	M	Renal pelvis	High	I	2.5cm×2cm×1.8cm	No	None	
UTUC-13_O	68	M	Renal pelvis	High	III	6cm×5.5cm×4cm	No	None	Success
UTUC-14_O	61	M	Renal pelvis	High	III	4cm×3cm×1.2cm	No	None	Success
UTUC-15_O	70	F	Renal pelvis	High	II	3cm×2.5cm×1cm	No	None	
UTUC-16_O	77	F	Ureter	Low	Ta	2cm×1cm×1cm	No	None	
UTUC-17_O	82	M	Renal pelvis	Low	I	1.5cm×0.7cm×0.3cm	No	None	Success
UTUC-18_O	66	M	Ureter	High	III	3.5cm×2cm×2cm	No	None	
UTUC-19_O	83	M	Renal pelvis	High	III	3.8cm×3cm×3cm	No	None	
UTUC-20_O	67	M	Ureter	Low	I	2cm×1.5cm×1cm	No	None	
UTUC-21_O	61	M	Ureter	High	I	2cm×2cm×1.7cm	No	None	
UTUC-22_O	86	F	Renal pelvis	High	II	4cm×2cm×1cm	No	None	
UTUC-23_O	69	M	Ureter	High	I	3cm×3cm×2.5cm	No	None	
UTUC-24_O	58	M	Ureter	High	I	3.2cm×1.5cm×1cm	No	None	
UTUC-25_O	45	M	Renal pelvis	Low	I	2cm×1.7cm×0.5cm	No	None	
Failed cases									
UTUC-26	62	F	Ureter	Low	I	3cm×2.2cm×0.8cm	No	None	
UTUC-27	63	M	Ureter	High	I	3.2cm×3cm×2cm	No	None	
UTUC-28	70	M	Renal pelvis	Low	I	1.5cm×1.5cm×1cm	No	None	
UTUC-29	67	M	Ureter	Low	I	3.5cm×2.5cm×2cm	No	None	
UTUC-30	81	F	Renal pelvis	High	III	6cm×5cm×3.5cm	No	None	
UTUC-31	65	M	Renal pelvis	High	II	1.3cm×1.2cm×0.1cm	No	None	
UTUC-32	57	F	Renal pelvis	High	II	1.2cm×0.8cm×0.3cm	No	None	

Table S2. UTUC organoid media recipe and basal medium recipe.

UTUC organoid media recipe			
Reagent Name	Supplier	Catalogue number	Final Concentration in Media
Advanced DMEM/F-12	Thermo Fisher Scientific	12634-010	1x
Antibiotic-Antimycotic (100X)	Thermo Fisher Scientific	15240-062	1x
GlutaMAX™ Supplement	Thermo Fisher Scientific	35050-061	1x
HEPES	Thermo Fisher Scientific	15630-080	10 mM
B-27™ Supplement (50X), serum free	Gibco	17504-044	1x
N-Acetylcysteine	Sigma	A9165	1.25 mM
Nicotinamide	Sigma	N0636	10 mM
A83-01	Sigma	SML0788	500 nM
Y-27632	Abmole Bioscience	M1817	10 mM
Basal medium (for the culture of normal organoids)			
Reagent Name	Supplier	Catalogue number	Final Concentration in Media
Advanced DMEM/F-12	Thermo Fisher Scientific	12634-010	1x
Antibiotic-Antimycotic (100X)	Thermo Fisher Scientific	15240-062	1x
GlutaMAX™ Supplement	Thermo Fisher Scientific	35050-061	1x
HEPES	Thermo Fisher Scientific	15630-080	10 mM
B-27™ Supplement (50X), serum free	Gibco	17504-044	1x
N-Acetylcysteine	Sigma	A9165	1.25 mM
Nicotinamide	Sigma	N0636	10 mM
SB202190	Sigma	S7076	10 mM
A83-01	Sigma	SML0788	500 nM
Recombinant Human R-Spondin 1 Protein	R&D Systems	4645-RS-250	500 µg/L
Human Noggin	Peprotech	120-10C-1000	100 µg/L
Y-27632	Abmole Bioscience	M1817	10 mM
Recombinant Human FGF-10	Peprotech	100-26	20 µg/L
Human EGF	Peprotech	AF-100-15	50 ng/mL

Table S3. List of antibodies used in this study.

Primary antibodies	Antigen	Supplier	Catalogue number	Origin	Reactivity	Dilution
	CK7	Thermo Fisher Scientific	180234	mouse	human	1/100
	p40	Maixin Biotech	RMA-0815	rabbit	human	1/100
	CK20	Life Technologies	12634-028	rabbit	human	1/100
	GATA3	Maixin Biotech	MAB-0695	mouse	human	1/100
	CK5	Thermo Fisher Scientific	RM-2106-S0	rabbit	human	1/100
	CD44	Cell Signaling Technology	17101-015	mouse	human	1/200
	UP II	NOVUS	NBP2-38904	rabbit	human	1/1000
	Ki67	Maixin Biotech	MAB-0672	mouse	human	1/100
Secondary antibodies	Reagent Name	Supplier	Catalogue number	Origin	Reactivity	Dilution
	Donkey anti-Rabbit IgG (H+L) Highly Cross-Adsorbed Secondary Antibody, Alexa Fluor 488	Thermo Fisher Scientific	A21206	donkey	rabbit	1/1000
	Goat anti-Mouse IgG (H+L) Cross-Adsorbed Secondary Antibody, Alexa Fluor 555	Thermo Fisher Scientific	A21422	goat	mouse	1/1000

Table S4. List of drugs used in this study.

Drug Name	Supplier	Catalogue number	Target	Max Concentration (μM)
Afatinib	Selleck Chemicals	S7810	EGFR/HER2	10 μM
AZD8055	Selleck Chemicals	S1555	mTOR	10 μM
AZD8931	Selleck Chemicals	S2192	EGFR, ErbB2, ErbB3	10 μM
Cabozantinib	Selleck Chemicals	S4001	c-Met/VEGFR2	10 μM
Cisplatin	Sigma	1134357	DNA crosslinker	100 μM
Crizotinib	Selleck Chemicals	S1068	ALK, c-Met	10 μM
Doxorubicin	Sigma	PHR1789	DNA replication	10 μM
Everolimus	Sigma	E-068 CERILLIANT	mTOR	10 μM
Fluorouracil	Selleck Chemicals	S1209	DNA replication	10 μM
Gemcitabine	Sigma	1288463	DNA replication	100 μM
Lapatinib	Selleck Chemicals	S2111	EGFR/HER22	10 μM
Methotrexate	Sigma	M9929	DHFR	100 μM
Mitomycin C	Selleck Chemicals	S8146	DNA crosslinker	10 μM
MK-2206	Selleck Chemicals	S1078	AKT1/2/3	10 μM
Paclitaxel	Selleck Chemicals	S1150	Tubulin	10 μM
Palbociclib	Selleck Chemicals	S1579	CDK4/CDK6	10 μM
PD173074	Selleck Chemicals	S1264	FGFR1	10 μM
Pemetrexed	Selleck Chemicals	S7785	TS, DHFR, GARFT	100 μM
SCH772984	Selleck Chemicals	S7101	ERK1/2	10 μM
Sorafenib	Selleck Chemicals	S7397	VEGFR/PDGFR	10 μM
Sunitinib	Selleck Chemicals	0	VEGFR2/PDGFR	10 μM
Temsirolimus	Selleck Chemicals	S1044	mTOR	10 μM
Thio-TEPA	Sigma	T6069	DNA crosslinker	100 μM
Trametinib	Selleck Chemicals	S2673	MEK1/2	10 μM

Table S5. List of primers used in this study.

Gene	Forward primer	Reverse primer

ALDH1A3	TGAATGGCACGAATCCAAGAG	CACGTCGGGCTTATCTCCT
CDK4	ATGGCTACCTCTCGATATGAGC	CATTGGGGACTCTCACACTCT
c-MET	GGTTCACTGCATATTCTCCCC	ACCATCTTTCGTTTCCTTTAGCC
RRM1	ACTTCGGCTTTAAGACGCTAGA	GCATGAGTAAACCACCTCTCAGA
